# HAK/KUP/KT family potassium transporter genes are involved in potassium deficiency and stress responses in tea plants (*Camellia sinensis* L.): expression and functional analysis

**DOI:** 10.1186/s12864-020-06948-6

**Published:** 2020-08-13

**Authors:** Tianyuan Yang, Xin Lu, Yan Wang, Yunxia Xie, Jingzhen Ma, Xunmin Cheng, Enhua Xia, Xiaochun Wan, Zhaoliang Zhang

**Affiliations:** State Key Laboratory of Tea Plant Biology and Utilization, Anhui Agricultural University, Hefei, 230036 Anhui China

**Keywords:** *Camellia sinensis*, HAK/KUP/KT family, K^+^ acquisition, Stress responses, Gene expression profiles

## Abstract

**Background:**

Tea plant is one of the most important non-alcoholic beverage crops worldwide. While potassium (K^+^) is an essential macronutrient and greatly affects the growth and development of plants, the molecular mechanism underlying K^+^ uptake and transport in tea plant root, especially under limited-K^+^ conditions, is still poorly understood. In plants, HAK/KUP/KT family members play a crucial role in K^+^ acquisition and translocation, growth and development, and response to stresses. Nevertheless, the biological functions of these genes in tea plant are still in mystery, especially their roles in K^+^ uptake and stress responses.

**Results:**

In this study, a total of 21 non-redundant *HAK/KUP/KT* genes (designated as *CsHAKs*) were identified in tea plant. Phylogenetic and structural analysis classified the *CsHAKs* into four clusters (I, II, III, IV), containing 4, 8, 4 and 5 genes, respectively. Three major categories of *cis*-acting elements were found in the promoter regions of *CsHAKs*. Tissue-specific expression analysis indicated extremely low expression levels in various tissues of cluster I *CsHAKs* with the exception of a high root expression of *CsHAK4* and *CsHAK5*, a constitutive expression of clusters II and III *CsHAKs*, and a moderate cluster IV *CsHAKs* expression. Remarkably, the transcript levels of *CsHAKs* in roots were significantly induced or suppressed after exposure to K^+^ deficiency, salt and drought stresses, and phytohormones treatments. Also notably, *CsHAK7* was highly expressed in all tissues and was further induced under various stress conditions. Therefore, functional characterization of *CsHAK7* was performed, and the results demostrated that CsHAK7 locates on plasma membrane and plays a key role in K^+^ transport in yeast. Taken together, the results provide promising candidate *CsHAKs* for further functional studies and contribute to the molecular breeding for new tea plants varieties with highly efficient utilization of K^+^.

**Conclusion:**

This study demonstrated the first genome-wide analysis of *CsHAK* family genes of tea plant and provides a foundation for understanding the classification and functions of the CsHAKs in tea plants.

## Background

Tea (the beverage) is made of bud and young leaves of tea plants (*Camellia sinensis*). Organic and inorganic components in young shoots determine the quality of the tea drink. Among these substances, catechins, theanine, and caffeine are the most important characteristic secondary metabolites in tea bud and leaf, which endow the tea with a rich taste and many health benefits [1–3]. Generally, the composition of the tea bud and young leaf is greatly influenced by many factors, such as the tea plant cultivar, nutrition status and environmental factors [4–7]. Out of these factors, mineral nutrition, especially nutrition of potassium, considerably affects the growth and development of tea plants [8]. For example, Ruan et al. (1998) reported that K^+^ fertilizer application increased the contents of free amino acids, caffeine and polyphenols in leaves of the various types of the tea plant. Additionally, exogenous K^+^ application or maintaining K^+^ accumulation in mesophyll cells appeared to mitigate remarkably the tea plant drought stress [9].

K^+^ is the most abundant cation in plant cells, accounting for 2 to 10% of plant dry matter. It is involved in many physiological processes including transmembrane transport, enzyme activation, anion neutralization, photosynthesis, osmoregulation, and stomatal movement [10, 11], thereby regulates plant growth, development and stress responses. Tea plants are preferably grown in tropical and subtropical regions where soil is usually highly leachy and acidic, and consequently, K^+^ deficient [12]. Inadequate application of K fertilizer often imposes many adverse impacts on tea plant, especially reduces photosynthetic efficiency and enzyme activity, and therefore causes a great loss of quality and yield of tea [13].

Due to the combinational effects of chemical fixation, root absorption, and loss by leaching, the K^+^ concentration in rhizosphere varies and rarely exceeds 1 mM [14]. Faced with the requirement of approximately 100 mM K^+^ concentration in plant cell cytoplasm for adequate metabolic function [10, 15], plants have evolved multiple K^+^ uptake systems in adaptation to this imbalance and variability [16–18]. Among these, HAK/KUP/KT (high-affinity K^+^ transporter / K uptake permease / K^+^ transporter) constitutes the largest gene family of plant K^+^ transporters which mediate K^+^ uptake at micromolar range [19, 20].

In the past decades, a series of plant *HAK/KUP/KT* genes were identified and their multiple physiological roles were characterized in many plant species [21, 22]. For instance, a total of 13 *HAK/KUP/KT* family members have been identified in the model plant *Arabidopsis* (*A. thaliana*) and 27 *OsHAKs* in rice (*Oryza sativa*). To date, with the release of genomes, HAK/KUP/KT family members have also been identified and characterized in other plant species, such as maize (*Zea mays*) [23], poplar (*Populus trichocarpa*) [24], tomato (*Solanum lycopersicum*) [25], soybean (*Glycine max*) [26] and pear (*Pyrus bretschneideri*) [27].

*HAK/KUP/KTs* express in different plant tissues, such as root [28], leaf [29], seed [30], and fruit [31]. Their expression is also highly regulated by K^+^ starvation [21], abiotic stresses including salt stress and drought [32–34], and phytohormones, such as, ABA (abscisic acid) [29], ethylene [35, 36], cytokinin [37] and NAA (1-naphthylacetic acid) [38]. These HAK/KUP/KT proteins demonstrated a great diversity of subcellular localization, such as plasma membrane [30, 32, 33], tonoplast [39], and endoplasmic reticulum [40]. Generally, they are shown to have K^+^ uptake activity by conferring the growth of K^+^-uptake-deficient bacteria or yeast mutants when they express in these mutants [28, 39, 41, 42]. Plant HAK/KUP/KT proteins typically contain 10–15 transmembrane (TM) segments and include a conserved K transport domain (GVVYGDLGTSPLY) [42–44]. They were generally classified into four clusters (I-IV) based on phylogenetic analysis [38, 39].

Expression patterns and physiological roles of some HAK/KUP/KTs from cluster I were characterized in both *Arabidopsis* and rice [17, 18, 20]. For example, the transcript level of *AtHAK5* was induced in root under K^+^-limitation conditions, while it was greatly down-regulated by high salinity [45]. It was further demonstrated that AtHAK5 played a key role in high-affinity K^+^ uptake under low K^+^ concentration even below 10 μM [46, 47]. In rice, *OsHAK1*, *OsHAK5* and *OsHAK21* were greatly induced in roots exposing to K^+^ deficiency and salt stress, and were shown to be involved in the maintenance of K^+^ homeostasis and salt tolerance [30, 32, 33]. Recently, Yang et al. (2020) showed that OsHAK5 is not only critical in K^+^ uptake, but also alters rice architecture by regulating ATP-dependent auxin transport [30, 48] OsHAK1 was recently shown to mediate K^+^ uptake under both low and high K^+^ concentrations and positively regulate drought stress response [34].

HAK/KT/KUPs from cluster II are proposed to mainly function in plant growth and development. In *Arabidopsis*, AtKUP4/TRH1 (Tiny Root Hairs 1) functions in maintaining polar localization of AtPIN1 in root apex and establishing appropriate auxin gradients, which in turn promote root gravitropism response and root hair formation [40, 49, 50]. Knockout of *AtKUP2/SHY3* (*Short Hypocotyl 3*) also caused developmental defects in shoots [51]. Notably, a triple null mutant *atkup2/6/8* showed promoted plant growth and impaired abscisic acid (ABA) responses in guard cells and lateral root cells [29].

Up to date, the information about the functions of *HAK/KUP/KT* genes from cluster III and IV is scarce [18, 20, 22]. Notably, however, *AtHAK7* was shown to be involved in K^+^ loading into xylem, and, consequently, in K^+^ transport from root to shoot, especially under K^+^-deficiency [52].

Due to the difficulty in genetic transformation and slow growth of tea plants, much fewer genes were identified and characterized in comparison to model plants. However, a rapid progress in tea biotechnology and functional genomics was achieved recently [53–55]. For example, Alagarsamy et al. (2020) reported that they firstly established an optimized method of Agrobacterium-mediated transgenic hairy root induction of *C. sinensis*. With this method, functional characterization of the “in root” genes could be carried out in vivo in the tea plant [56]. More recently, a native *C. sinensis* Tannase gene (CsTA) was identified for the first time in plant kingdom [57]. The transcriptional and metabolic analyses revealed that the expression of CsTA negatively regulated the accumulation of galloylated catechins [57]. Moreover, Dong et al.(2020) showed that six amino acid permeases in tea plant exhibited theanine transport activity, and suggested a role for CsAPP1 in transporting theanine from roots toward new shoots [58]. Such progress related to tea plant biotechnology should contribute to a better understanding of the tea plant biological characteristics.

The study of the molecular underpins of the tea plant K^+^ nutrition is also bound to benefit from the biotechnological progress. Although K^+^ is one of the most essential macronutrients, its uptake and transport has not been studied in molecular level in the tea plants. HAK/KUP/KT family has not been systematically identified in tea plant, either [55]. With the release of the tea plant genomes [59, 60], we currently have an opportunity to systemically identify HAK/KUP/KT family and investigate their physiological roles. In this study, 21 non-redundant *CsHAKs* were identified in the tea plant. Subsequently, gene structures, phylogenetic relationships, tissue-specific expression, and expression in responses to K^+^ deficiency and various stresses were investigated. To further reveal the role of HAK/KUP/KTs in the tea plant, the subcellular localization and functional characterization of CsHAK7 in K^+^ acquisition were also performed. Overall, our results provide a foundation for further functional characterization of HAK/KUP/KTs in the tea plant and contribute to a better understanding of the molecular basis of *CsHAKs’* responses to K^+^ deficiency and various stresses.

## Results

### Identification and sequence analysis of *CsHAKs* in the tea plant

To identify the complete set of CsHAK family members in the tea plant genome, the sequences of 13 *Arabidopsis* and 27 rice HAK/KUP/KT proteins were used as queries to screen the local tea plant genome database. Then, the HMM profile of the HAK domain (K_trans; PF02705) was performed to conduct a global search of the tea plants genome [60]. Next, both Pfam database and InterProScan database were used to further confirm the presence of the conserved HAK domain. A total of 21 non-redundant *CsHAKs* were identified in the tea plant genome (Table 1). Due to the different number of *CsHAKs* between tea plant and Arabidopsis, therefore, we named some of these CsHAKs based on their homology with those of Arabidopsis, and the reminding are named freely. The detailed information on the *CsHAKs* (including gene name, sequence ID, protein length, molecular weight, theoretical isoelectric point, predicted subcellular localization) has been listed in Table 1. The predicted CsHAK proteins had considerably different amino acid lengths and molecular weights. Their lengths ranged from 691 to 947 amino acid residues. The values of their theoretical isoelectric points varied from 5.36 to 9.28. CsHAK17 had the lowest molecular weight (77.28 kDa) and CsHAK14 had the highest (105.26 kDa). Based on the prediction by TMME2.0 web server, the number of transmembrane segments (TMS) of CsHAKs ranged from 10 to 14 (Fig. S1). The majority of CsHAKs were predicted to localize to the plasma membrane, with several of them probably in the cytoplasm and chloroplast (Table 1).

**Table 1:** Information of CsHAKs identified in tea plants

Name	Sequence ID	Protein											
		Length (aa)	pI	MW (KDa)	Subcellular localization	B	FL	FR	L1	L2	L3	R	S
CsHAK1	TEA008833.1	714	8.10	80.03	Plasma membrane	110.9029	42.0600	11.2404	44.7742	3.8711	0.6120	14.1058	42.7326
CsHAK2	TEA022010.1	802	8.48	89.68	Cytoplasm	27.9207	64.8636	21.2298	18.7921	1.9196	1.5295	3.1587	21.1625
CsHAK3	TEA005066.1	787	8.64	87.40	Plasma membrane	51.2697	45.7785	59.9698	91.1863	85.0903	120.2709	68.3359	83.5590
CsHAK4	TEA003872.1	752	7.25	84.26	Plasma membrane	0.2042	0.1092	0.3867	0.1139	0.0656	0.0222	0.7034	0.1293
CsHAK5	TEA003873.1	809	8.47	91.38	Plasma membrane	0.0072	0.3697	0.0249	0.0075	0.0000	0.0100	18.7464	0.0085
CsHAK6	TEA011201.1	767	8.60	86.69	Cytoplasm	19.8532	17.0587	50.9855	40.9706	16.1786	73.3125	9.4973	31.0653
CsHAK7	TEA003094.1	908	5.74	101.11	Plasma membrane	11.5103	10.8720	13.0813	9.8839	13.4190	12.8203	10.8639	16.6949
CsHAK8	TEA007724.1	805	7.32	90.78	Plasma membrane	10.3956	9.6716	18.6232	17.7413	14.0597	18.1328	7.5946	20.9987
CsHAK9	TEA003884.1	736	9.28	82.90	Plasma membrane	0.1850	0.1677	0.0461	0.1167	0.1727	0.1268	0.1135	0.0378
CsHAK10	TEA021417.1	855	7.32	95.86	Plasma membrane	47.0292	49.8357	25.9564	44.7135	39.1111	30.2252	70.2434	36.9693
CsHAK11	TEA006987.1	732	8.67	80.32	Plasma membrane	1.8167	1.9706	1.4882	1.8318	4.0013	3.7068	2.6004	3.9919
CsHAK12	TEA032656.1	709	5.52	79.24	chloroplast	15.6754	9.6164	17.9050	13.8896	20.9087	10.2305	14.5307	20.7014
CsHAK13	TEA023021.1	734	8.81	81.51	Plasma membrane	0.0645	41.9444	1.4596	0.1589	4.9184	0.8987	0.3129	2.4945
CsHAK14	TEA011420.1	947	8.93	105.26	Plasma membrane	0.6758	1.5437	0.1622	0.4367	0.4758	0.4585	0.1869	12.1504
CsHAK15	TEA012915.1	710	8.95	79.08	Plasma membrane	1.0015	2.3787	1.4008	0.5114	3.1257	0.4787	0.5389	10.8703
CsHAK16	TEA016241.1	703	8.33	78.77	Plasma membrane	0.0085	67.3102	0.0388	0.0000	0.0000	0.0356	0.2088	0.0299
CsHAK17	TEA018688.1	691	7.57	77.28	Plasma membrane	5.3419	19.1077	5.2523	7.2408	7.3490	21.1981	28.6248	14.8871
CsHAK18	TEA023299.1	841	5.36	93.86	Plasma membrane	6.2981	20.2273	4.1755	5.4650	8.4885	10.9642	7.6997	8.8804
CsHAK19	TEA025917.1	780	8.57	86.17	Plasma membrane	0.1056	0.2360	0.0605	0.0156	0.0810	0.0634	4.5690	0.1330
CsHAK20	TEA031103.1	702	8.43	79.09	Plasma membrane	0.0254	0.0294	0.0000	0.0439	0.0000	0.0000	0.0000	0.0000
CsHAK21	TEA032638.1	789	7.55	87.77	Plasma membrane	3.9471	4.2648	9.7922	2.2762	1.2882	25.0887	3.5484	3.3271

### Phylogenetic analysis of CsHAKs

**Fig. 1:**
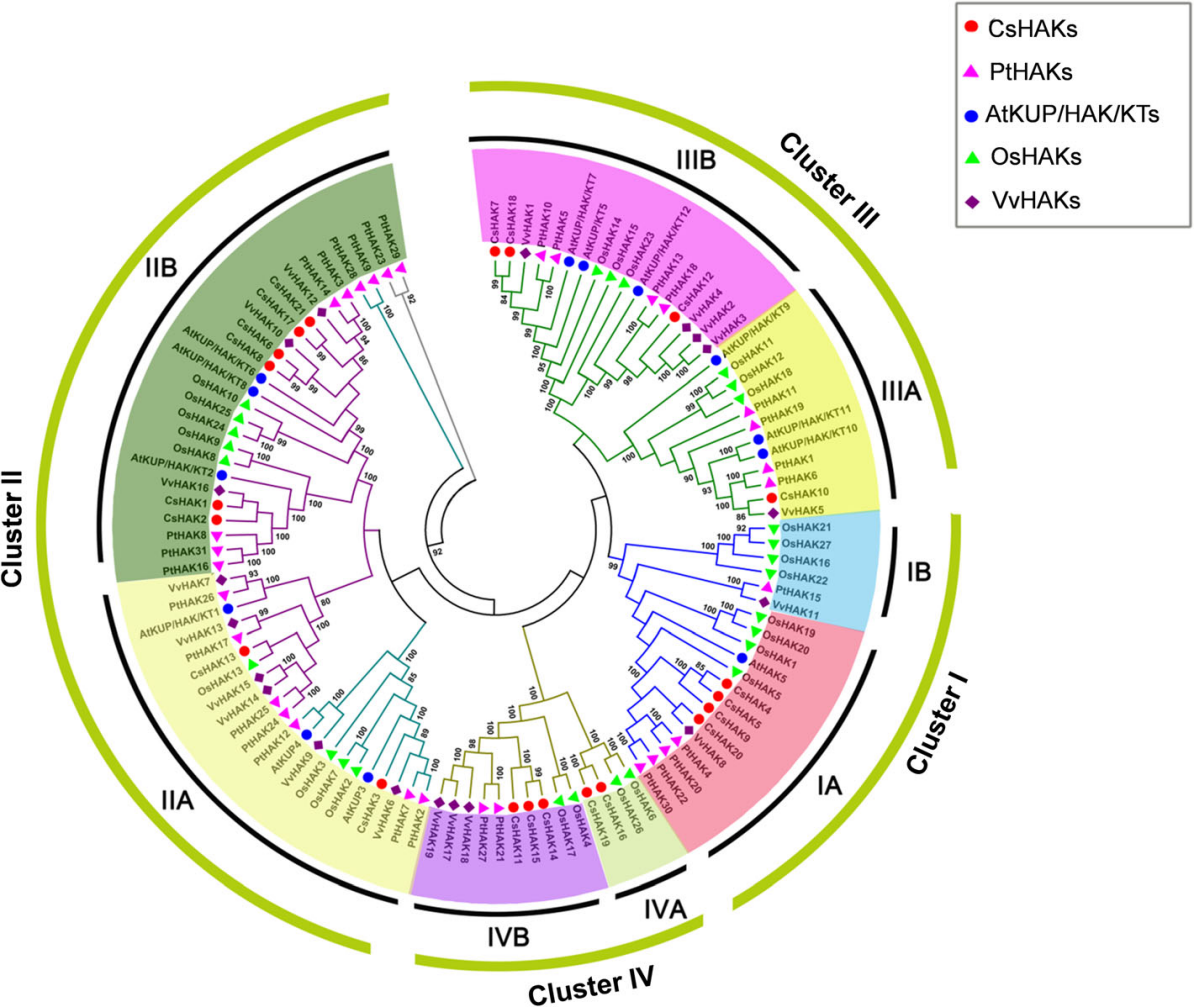
Phylogenetic tree of KT/HAK/KUP family proteins among tea plant, poplar, rice, grape and *Arabidopsis*. The proteins belonging to each of the five species are represented by different colors and shapes. The KT/HAK/KUP family proteins were classified into four clusters (I, II, III, IV). Every cluster further were divided into two subgroups including cluster IA and IB, Cluster IIA and IIB, Cluster IIIA and IIIB, Cluster IVA and IVB, based on their sites in a same branch. The gene ID of tea plant, poplar, rice, grape and Arabidopsis KUP/HAK/KT family proteins are listed in Table S2. Cs, *Camellia sinensis*; Pt, *Populus trichocarpa*; At, *Arabidopsis thaliana*; Os, *Oryza sativa* .Vv, *Vitis vinifera*

To reveal the evolutionary relationship and functional divergence of the CsHAK family members, the full length of 111 HAK proteins, including 21 from tea plants, 13 from Arabidopsis, 27 from rice, 31 from poplar and 19 from grape, were used to construct a phylogenetic tree (Fig. 1). These HAK/KUP/KT proteins were classified into four clusters, with 4, 8, 4, 5 members, in clusters I, II, III and IV, respectively (Fig. 1). Clusters II and IV members were the most abundant in tea plants, comprising 61.90% of all CsHAKs. Importantly, these proteins, including CsHAK4, 5, 9 and 20, were distributed in cluster I together with the already-characterized AtHAK5 [46], OsHAK1 [32, 34] and OsHAK5 [30, 62], suggesting that they also have a crucial role in the tea plant K^+^ uptake from a low-K^+^ soil. Among the cluster II members, eight of the CsHAKs (CsHAK1, 2, 3, 6, 8, 13, 17, 21) were found together with AtKUP2 [51] and AtKUP4 [40, 49, 50], implying that they are likely involved in developmental processes in the tea plant. Notably, among members of cluster III, CsHAK7 shared the almost highest sequence identity (77.9%) with AtKUP7 [52], suggesting a role for CsHAK7 in K^+^ acquisition and translocation in the tea plant root in the presence of a low K^+^ concentration.

### Gene structure and conserved motifs of CsHAKs

**Fig. 2:**
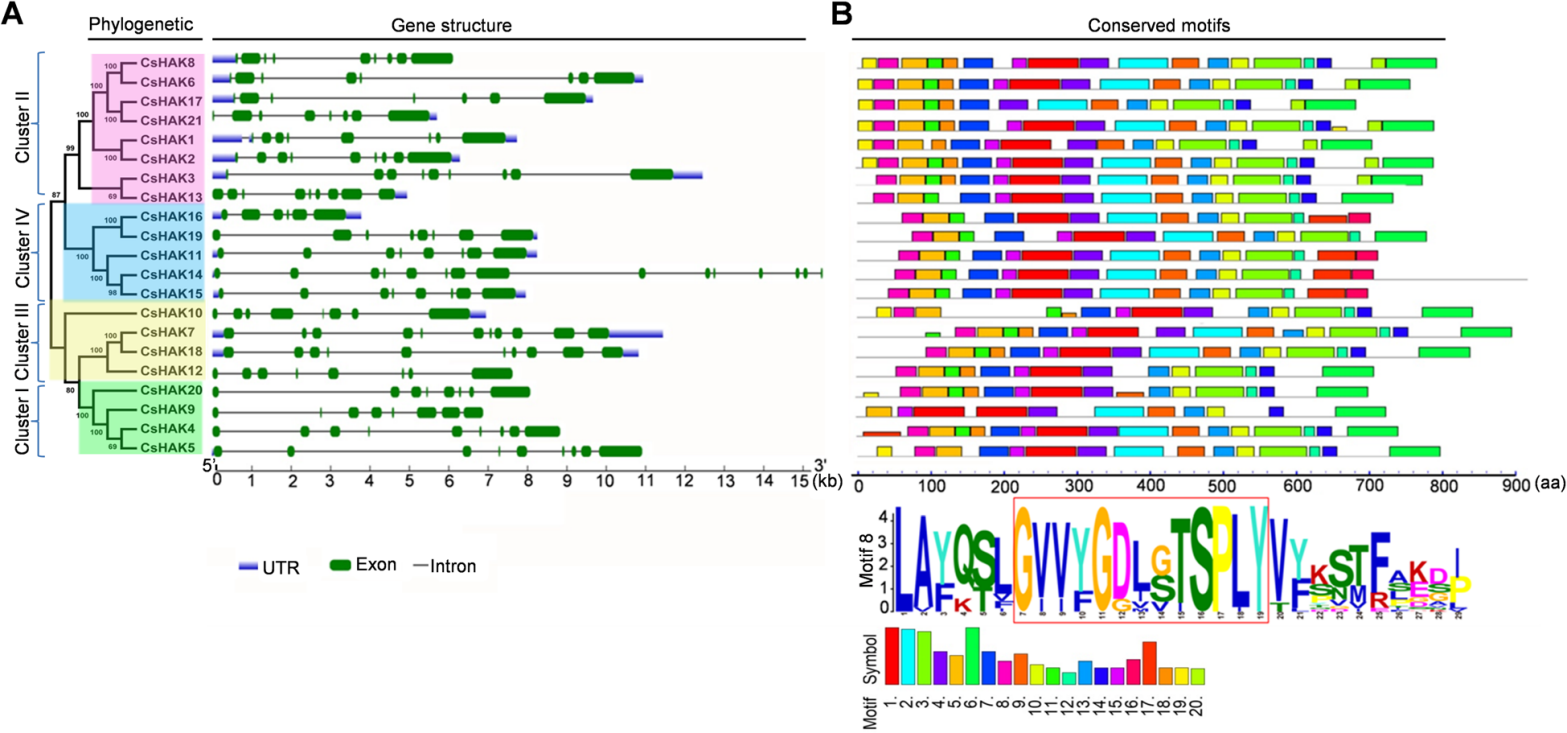
Phylogenetic tree, exon-intron structure and distribution of conserved motifs structure of the *CsHAKs*. **a** *CsHAKs* are displayed in order based on a phylogenetic analysis of their protein products. Introns, exons and noncoding regions are displayed with black lines, blue boxes and green boxes, respectively. **b** Distribution of twenty motifs in CsHAKs are marked by different colors of boxes and conservation of motif 8 was present all tea plant HAK proteins

To gain insights into the evolutionary relationships and structural features, the exon/intron structures of *CsHAKs* and conserved motifs in CsHAKs were investigated. CsHAK proteins in cluster I-IV were listed in order based on the phylogenetic analysis (Fig. 1 and Fig. 2a). The gene structure of *CsHAKs* was constructed by comparing the CDS and genomic sequences using an online gene structure display server (GSDS) 2.0 program. Most of members of *CsHAKs* possessed 6 to 10 exons and 5 to 9 introns except for *CsHAK14* (15 exons /14 introns) (Fig. 2a). As expected, most of *CsHAKs* belonging to the same cluster also displayed similar distribution patterns of exon/intron in terms of exon length and intron number (Fig. 2a). This was in agreement with the results reported in rice [38] and wheat [63].

To investigate the distribution of conserved motifs in CsHAKs, a total of 20 conserved motifs were identified by the MEME program and were designated as motifs 1–20. The highly conserved motif containing a special K^+^ transport domain (GVVYGDLGTSPLY) was emphasized with motif logo (motif 8) (Fig. 2b), and it existed in all CsHAK proteins (Fig. 2b; Table S4). Generally, the 20 motifs were almost evenly distributed in CsHAK proteins. Motifs 1, 2, 4, 5, 7 and 8 possessing feature domain of K^+^ transporter (Fig. 2b, Table S3) were observed in all CsHAK proteins. Together, CsHAK proteins belonging to the same cluster exhibited a similar motif distribution, which was consistent with the results of gene structure analysis.

### Analysis of *cis*-elements in the *CsHAKs* promoters

**Fig. 3:**
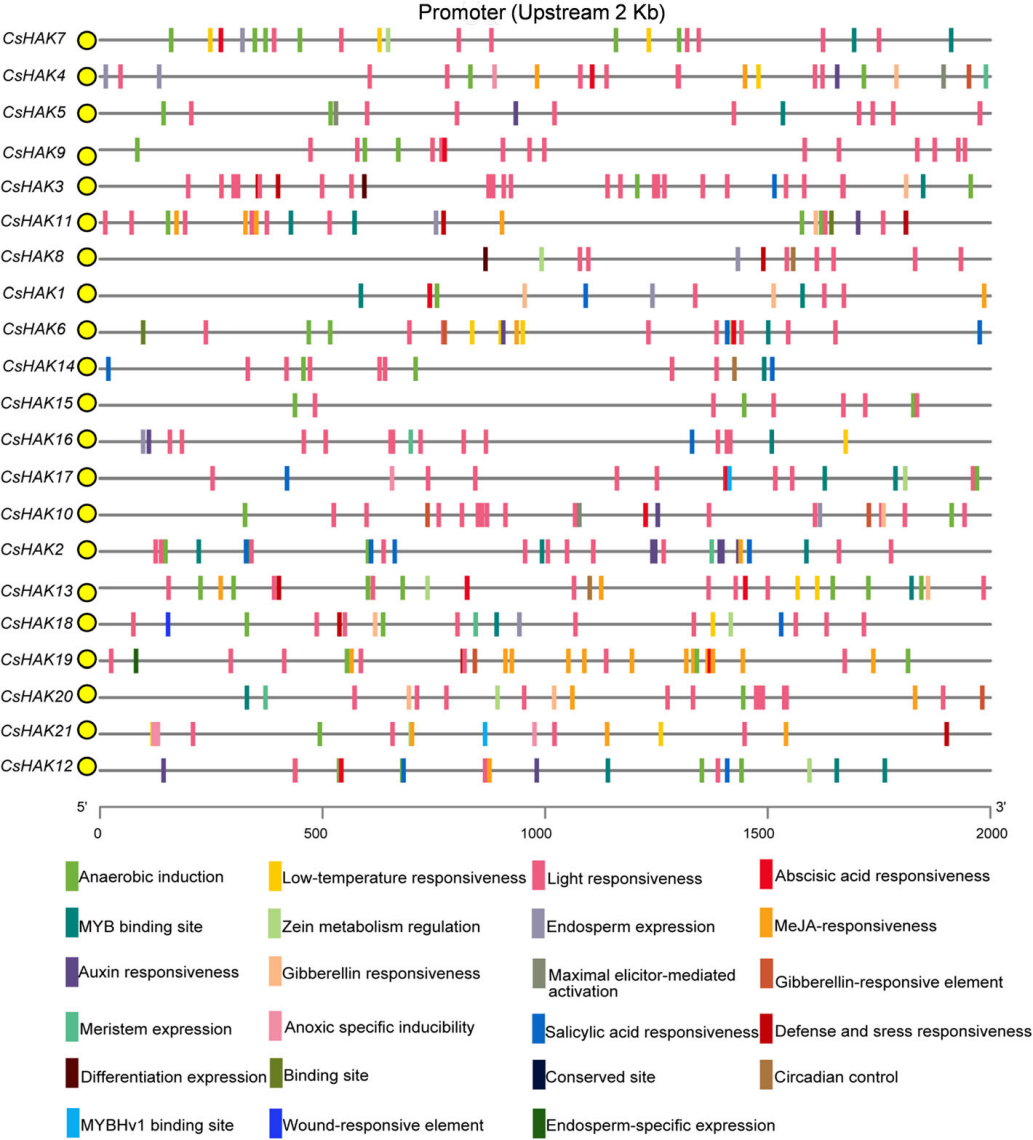
*Cis*-acting elements in the promoter regions of *CsHAKs*. Based on the functional annotation, these *cis*- acting elements were classified into three major categories (including plant growth and development responses, phytohormone responses and stresses-related responses). Detailed *cis*- acting elements positions in the promoters of *CsHAKs* are listed in Supplementary Table S6

To understand the transcriptional regulation and function of *CsHAKs*, the promoter regions (2000 bp upstream of the transcription start site) were used to identify *cis*-elements using the PlantCARE database (Fig. 3, Fig. S2, Table S5). A total of twenty-three types of *cis*-acting elements were identified. These elements randomly distributed in the promoter regions of *CsHAKs* and were predicted to participate in plant growth and development responses, stress and phytohormone responses. Among the *cis*-acting elements related to plant growth, eight endosperm expression regulatory elements and eight zein metabolism regulation element were found in the promoters of *CsHAKs*. The CAT-box involved in meristem expression was identified in the promoters of five *CsHAKs*. Among the *cis*-acting elements belonging to hormone responses, auxin-, ethylene-, abscisic acid-, the MeJA- and gibberellin-responsive elements were observed in the promoters of eight, twenty, eleven, ten and eight *CsHAKs* (Fig. 3, Fig. S2, Table S5), respectively. Among stress-related responses elements, interestingly, anaerobic induction element (ARE element) was observed in the promoters of almost all *CsHAKs* except for *CsHAK8*. Moreover, stress-related (defense and wound, stress response to low-temperature and drought) *cis*-acting elements were also identified in the promoters of seven, one, seven and twelve *CsHAKs* (Fig. 3, Fig. S2, Table S5). Also, *cis*- acting elements responsive to one of the most important environmental factors, light were present in the promoters of all *CsHAKs* (Fig. 3, Fig. S2, Table S5).

### Tissue-specific expression of *CsHAKs*

**Fig. 4:**
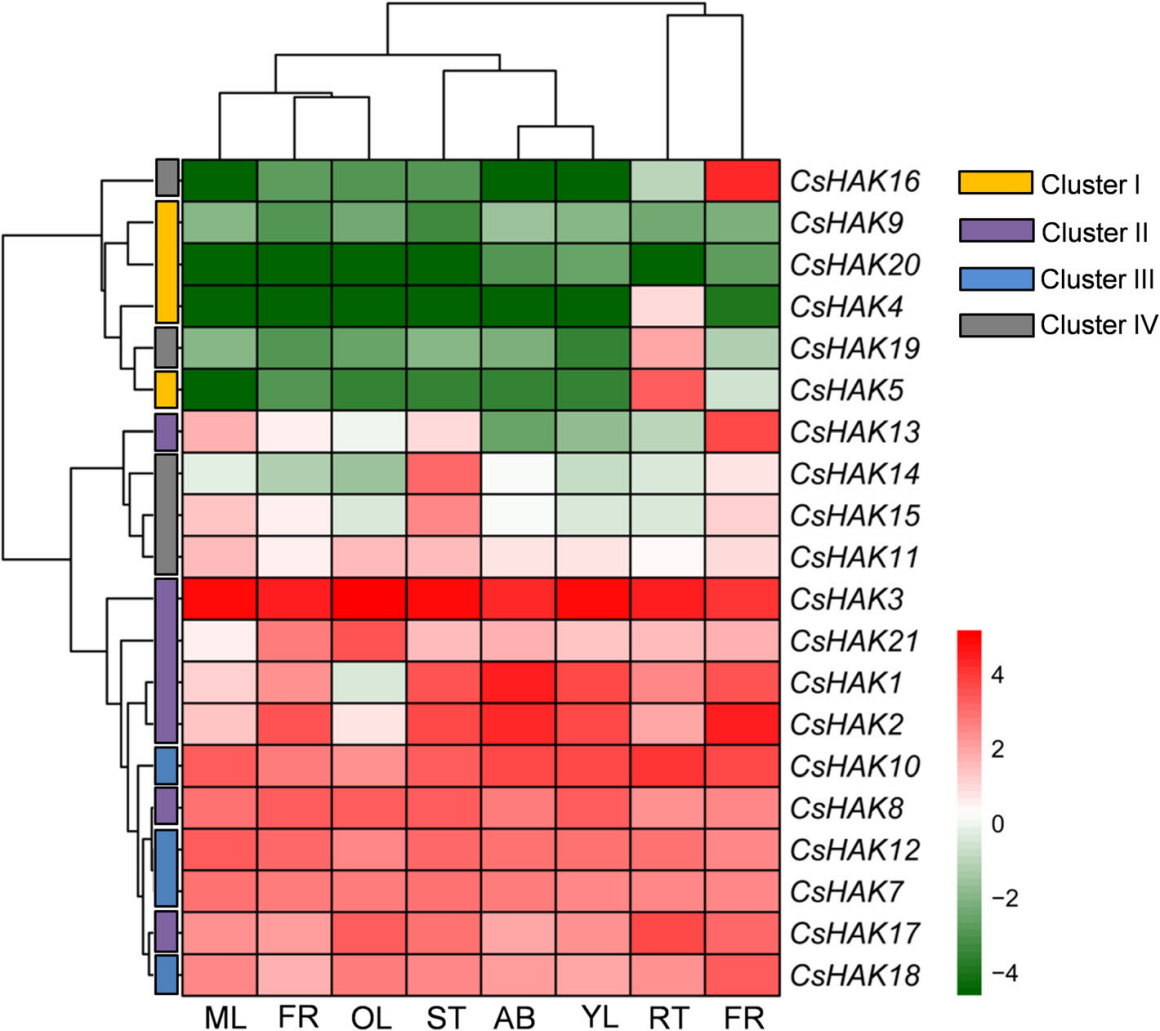
Expression patterns of *CsHAKs* in different tea plant tissues. The color bar represents Log_2_FPKM. Red represents high expression level, green represents low expression level. The members of the *CsHAKs* belonging to the four clusters are represented by rectangles of different colors. The 8 tissues are apical buds (AB), young leaf (YL), mature leaf (ML), old leaf (OL), young stem (ST), flower (FL), young fruit (FR) and tender root (RT). Detailed FPKM values are listed in Table S5

To further unveil the potential roles of *CsHAKs*, the tissue-specific expression of all the *CsHAKs* was analyzed using reliable transcriptomic database of 8 tissues of tea plant [59]. Importantly, the organs/tissues represented different developmental stages of the tea plant (Fig. 4). Hierarchical clustering was generated using the FPKM (fragments per kilobase million) values of eight tissues (detailed FPKM values are listed in Table S6) to present the relative expression levels of *CsHAKs* in different tissues. As shown in Fig. 4, some *CsHAKs* showed similar expression levels in the eight tissues, while other *CsHAKs* presented significant tissue-specific expression patterns, suggesting the functional divergence of *CsHAKs* among the tea plant tissues during growth and development. For example, *CsHAK3* was constitutively and highly expressed in all tissues, while *CsHAK9* and *CsHAK20* were expressed at extremely low levels in all tissues (Fig. 4, Table S6). Notably, *CsHAK4*, *CsHAK5*, and *CsHAK19* were preferentially expressed in the root, implicating them in K^+^ acquisition from the soil (Fig. 4, Table S6). *CsHAK16* was highly expressed in the flower, suggesting *CsHAK16*’s involvement in flower development (Fig. 4, Table S6). In addition, *CsHAK1* and *CsHAK2* showed relatively high expression in the apical bud and young leaves, suggesting they have an important role in young shoot growth. *CsHAK14* and *CsHAK15* expression in the stem was relatively high, implying a possible participation in long-distance transport of K^+^ in the tea plant. It is noteworthy that some *CsHAKs* (*CsHAK7*, *8*, *10*, *12*, *17*, *18*) maintained a substantially high expression in all tissues (Fig. 4, Table S6), suggesting that these genes play an important role in all these tissues represented. Increasingly, studies on different plant species suggested that *HAK/KUP/KTs* from cluster I are mainly expressed in root and play an essential role in high-affinity K^+^ transport in plant [30, 32, 33], while HAKs from cluster II were shown to be involved in plant growth and development and were expressed in almost all plant tissues [29, 40]. Based on these and similarity in expression patterns of *CsHAKs* in tea plant, we speculated that CsHAKs from the same cluster perform similar functions.

### The effect of K^+^ deficiency on *CsHAKs* expression

**Fig. 5:**
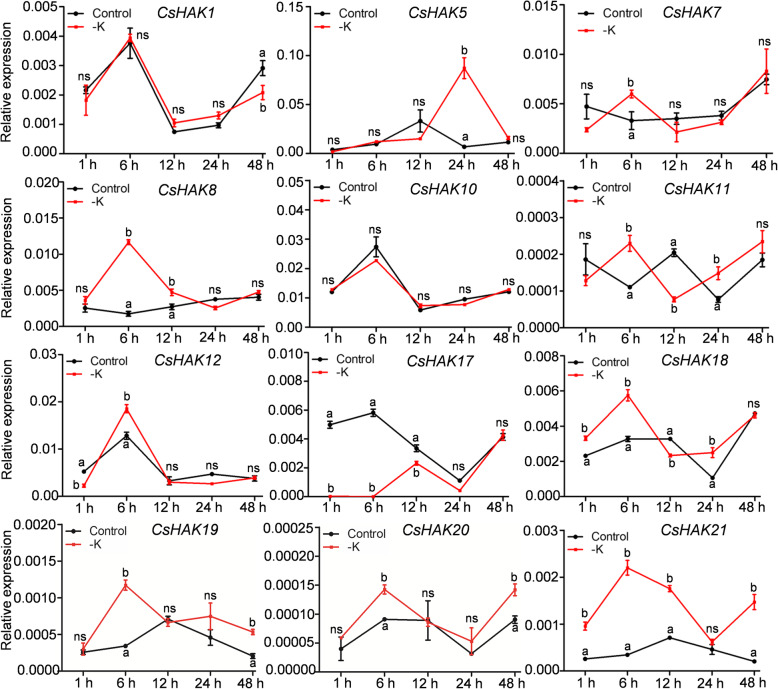
Expression profiles of *CsHAKs* in the tea plant root in response to K^+^ deficiency. *CsGAPDH* was an internal control. The mean value was calculated from three independent replicates. Error bars indicate standard errors of three biological replicates. Significant differences from the control in each group are indicated by different letters (*P* < 0.05, one-way ANOVA). ns, no siginificance

It has been extensively reported that the HAK/KUP/KT transporters are essential for K^+^ uptake and long-distance transport in various plant species, especially under K^+^-limiting conditions [20, 64]. To examine responses of *CsHAKs* to K^+^ deficiency, the transcript levels of twelve selected *CsHAKs* from four clusters (including cluster I: *CsHAK5*, *20*; cluster II: *CsHAK1*, *8*, *17*, *21*; cluster III: *CsHAK7*, *10*, *12*, *18* and cluster IV: *CsHAK11*, *19*) were assessed using qRT-PCR in the tea plant roots exposed to K^+^ deficiency. On the whole, the expression levels of eight *CsHAKs* were induced by K^+^ removal compared to control condition. However, three different expression patterns were found among these genes. The first pattern was characterized by rapid upregulation by K^+^ removal, as seen in *CsHAK7*, *8*, *11*, *12*, *18*, *19*, *20* (Fig. 5). The second pattern is exemplified by *CsHAK21*, whose expression level of which remained continually elevated during K^+^ deficiency (as assayed at the indicated time points), except at 24 h (Fig. 5). The third pattern was exemplified by *CsHAK5*. Its expression level barely changed till 12 h after K^+^ starvation, followed by a remarkable elevation at 24 h, comparing to the control condition (Fig. 5). In contrast, the expression of *CsHAK17* was constantly suppressed in the absence of K^+^ at all time points up to 12 h. No significant change was observed in the expression levels of *CsHAK1* and *CsHAK10* after removal of K^+^. These results demonstrate that among the tested *CsHAKs, CsHAK7*, *8*, *11*, *12*, *18*, *20* were highly expressed in roots, greatly responsive to K^+^ deficiency and could be involved in K^+^ acquisition from soil by tea plants.

### Effects of various stresses and phytohormones on the expression of *CsHAKs*

In addition to grow in plains, most tea plants grow in the wild in mountainous and hilly areas and often face potassium starvation. Generally, K^+^-starved tea plants are greatly vulnerable to various stress types, both biotic (pests, fungal diseases) and abiotic (drought, cold, high temperature) [9, 61, 65]. Potassium improves the resistance of tea plants to these stresses and promotes the synthesis and partitioning of photosynthate [8, 12, 65]. Therefore, we further examined the response of *CsHAKs* expression in root to abiotic stresses and phytohormones.

**Fig. 6:**
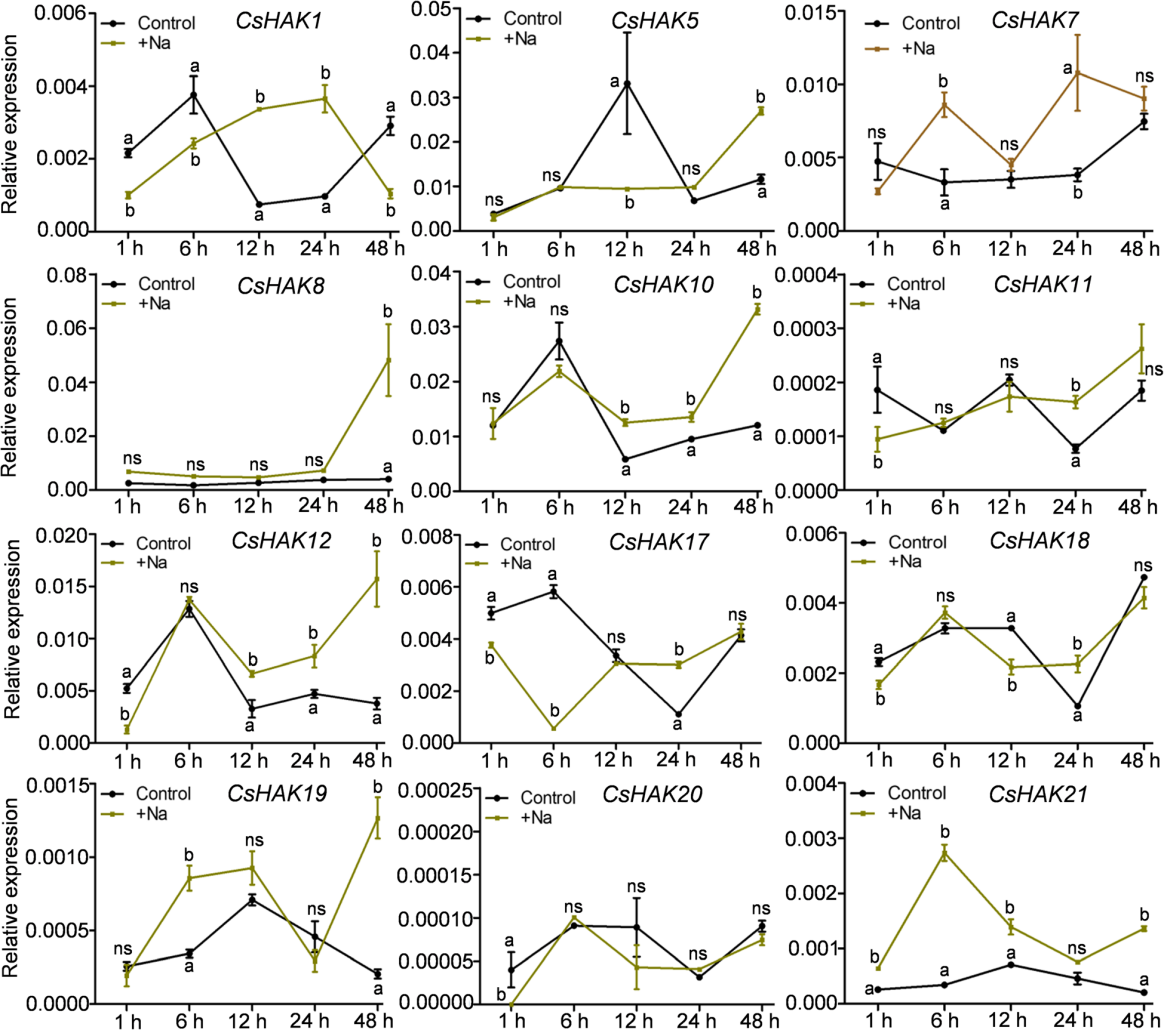
Expression profiles of *CsHAKs* in tea plant root in response to salt stress. *CsGAPDH* was an internal control. The mean value was calculated from three independent replicates. Error bars indicate standard errors of three biological replicates. Significant differences from the control in each group are indicated by different letters (*P* < 0.05, one-way ANOVA). ns, no siginificance

*HAK/KUP/KT* gene expression was strongly induced by salt stress, and played a crucial role in enhancing salt tolerance in other plants [30, 66]. Similarly, most of tested *CsHAKs*, including *CsHAK1*, *5*, *7*, *8*, *10*, *11*, *12*, were appreciably upregulated in the presence of salt stress (Fig. 6). In contrast, the expression of *CsHAK17* was greatly suppressed, while that of *CsHAK11*, *CsHAK18* and *CsHAK20* remained stable at the same conditions (Fig. 6).

**Fig. 7:**
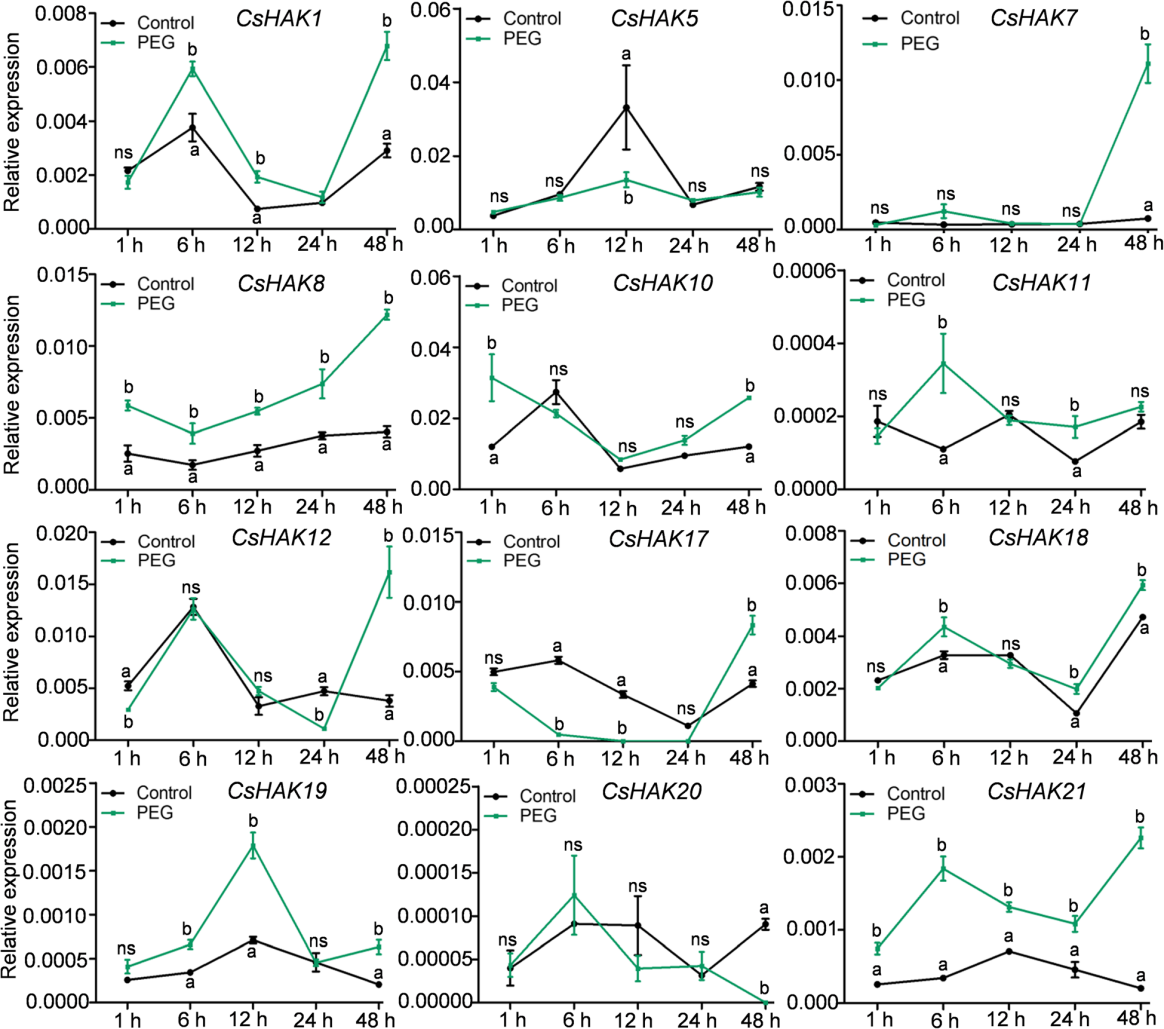
Expression profiles of *CsHAKs* in tea plant roots in response to drought stress. *CsGAPDH* was an internal control. The mean value was calculated from three independent replicates. Error bars indicate standard errors of three biological replicates. Significant differences from the control in each group are indicated by different letters (*P* < 0.05, one-way ANOVA). . ns, no siginificance

Drought is a common environmental stress of the tea plant during the whole growth and development period [9]. To reveal the potential role of CsHAKs in response to dehydration, drought stress was mimicked by applying 20% PEG6000 in the culture solutions. The expression of most of *CsHAKs* increased at different time points since the start of “drought” exposure, albeit in varying patterns (Fig. 7). *CsHAK7* and *CsHAK12* showed similar expression pattern and reached maximum at 48 h after PEG6000 treatment. In contrast, the expression of *CsHAK5*, *CsHAK18* and *CsHAK20* did not significantly respond to drought stress. Additionally, the expression level of *CsHAK17* was continuously down-regulated during the 24 h of exposure to the treatment, and was then moderately upregulated at 48 h (Fig. 7).

**Fig. 8:**
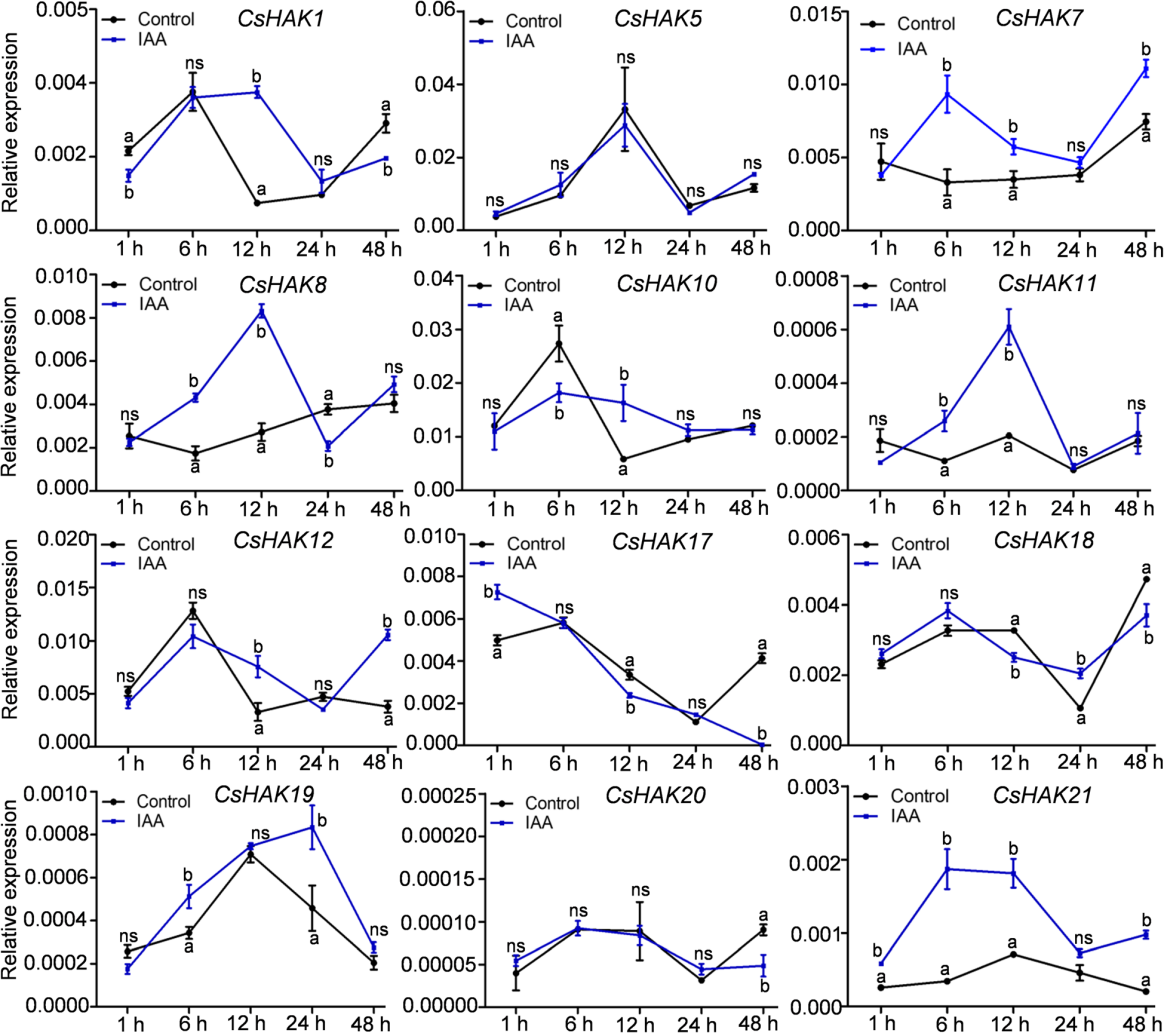
Expression profiles of *CsHAKs* in tea plant roots in response to IAA treatment. *CsGAPDH* was an internal control. The mean value was calculated from three independent replicates. Error bars indicate standard errors of three biological replicates. Significant differences from the control in each group are indicated by different letters (*P* < 0.05, one-way ANOVA). ns, no siginificance

Previously studies showed that *HAK/KUP/KT* expression was regulated by phytohormones in plants [29, 38]. Therefore, we investigated the effects of IAA and ABA on the expression of *CsHAKs*. Under IAA treatment, the expression of *CsHAK1*, *7*, *8*, *11*, *12*, *21* was markedly elevated, while the remaining *CsHAKs* showed no obvious induction of expression except for one or two time points (Fig. 8). The *cis*-acting element of auxin responsiveness was found in the promoters of *CsHAK11* and *CsHAK12.* This element is likely to be involved in regulating *CsHAK11* and *CsHAK12* expression in response to IAA treatment. ABA treatment increased the expression of five *CsHAKs* (*CsHAK7*, *8*, *12*, *18*, *19*) compared to controls and did not affect the expression of other *CsHAKs* (Fig. S3).

Abiotic stresses, such as salt and drought, are generally increased the accumulation of ABA [67]. The presence of multiple *cis*-acting elements of ABA responsiveness which we found in the promoters of the tested genes (Fig. 3) correlated with the effect of ABA on these genes expression. In addition, *CsHAK7*, *8*, *11*, *12* have IAA responsiveness elements in their promoters and were also responsive to IAA treatments. These results suggested a lot of these cis-elements in the regulation of CsHAKs by abiotic stresses, ABA and IAA.

### Functional characterization and subcellular localization of CsHAK7

**Fig. 9:**
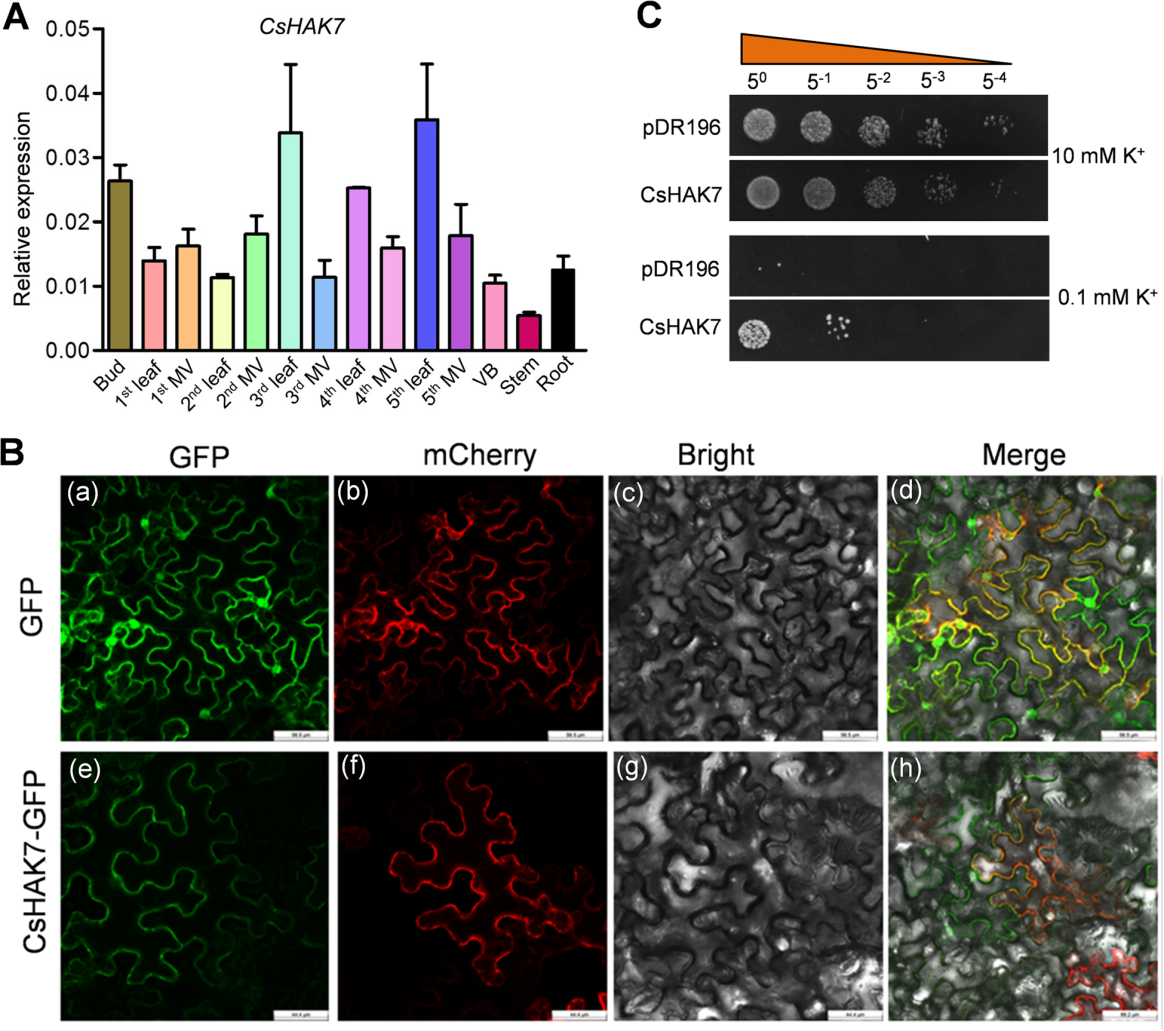
Tissue-specific expression pattern, subcellular localization and yeast complementation analysis of CsHAK7. **a** Expression pattern of *CsHAK7* in indicated tissues of tea plant. MV, major vein; VB, vascular bundle, peeled from the stem. *CsGAPDH* was an internal control. Data are mean ± SE (*n* = 3). **b** Confocal laser scanning microscopy images showed tobacco leaf epidermal cells transiently expressing either GFP or CsHAK7::GFP together with AtPIP2A:mCherry (plasma membrane maker, Nelson et al., 2007). (a), (e), Confocal images via the GFP channel only. (b), (f), Confocal images of the red mCherry fluorescence marking the PM position. (c), (j), Bright field. (d), (h), Merged images of GFP (green) and mCherry RFP (red) together with bright filed. Scale bar, 45 μm. **c** Yeast complementation assay of K^+^ acquisition by the K^+^ uptake-defective yeast mutant (R5421) complemented with CsHAK7. Growth status of R5421 cells expressing CsHAK7, empty vector (pDR196), on AP solid medium containing 10 or 0.1 mM K^+^. The 1:5 serial dilutions of yeast cells were spotted on the AP solid medium and then incubated at 30 °C for 3–5 d

We selected CsHAK7 for further functional characterization considering the following facts. Firstly, *CsHAK7* was constitutively expressed in all eight tissues tested. Secondly, *CsHAK7* expression was induced by K^+^ deficiency, salt and drought stresses, IAA and ABA treatments. Thirdly, within the 21 CsHAKs, CsHAK7 has the relative higher identity with the homolog in Arabidopsis (Table S2), and AtHAK7 has been well functionally characterized [52]. These facts suggested a critical role of CsHAK7 in tea plant growth and stress responses. To confirm the expression patterns of *CsHAK7* in the eight tissues obtained by RNA-seq, we verified its expression patterns by qRT-PCR. As shown in Fig. 9a, *CsHAK7* was constitutively and highly expressed, although not at the same level, in all the tissues tested, especially in mature leaves and buds. This was generally consistent with the transcriptome data (Fig. 4).

CsHAK7 was predicted to be located in the plasma membrane (Table 1). To verify its subcellular localization, the whole ORF without the stop codon of *CsHAK7* was fused to the N-terminal of GFP reporter of pCAMBIA1305 expression vector driven by CaMV 35S promoter, generating a fusion construct *CsHAK7::GFP*. The *CsHAK7::GFP* and *AtPIP2A::mCherry* (plasma membrane marker gene) [68] were co-transformed into tobacco leaf epidermal cells. Microscopic visualization demonstrated that the fluorescence of CsHAK7-GFP overlapped with the fluorescence of *AtPIP2A::mCherry* (Fig. 9b). However, green fluorescence was observed in the entire cell region when the only GFP plasmid was transformed into tobacco leaf epidermal cells (Fig. 9b). The results demonstrated that CsHAK7 is localized in the plasma membrane.

To characterize the function of CsHAK7 in K^+^ uptake, a yeast mutant complementation assay was performed. Initially, the plasmids of yeast empty vector pDR196 and pDR196-CsHAK7 were transformed into a K^+^-uptake-defective strain mutant R5421 [30]. This mutant could not grow well in the presence of low concentration of K^+^. The yeast growth assays were conducted on solid arginine phosphate (AP) medium containing high or low K^+^ concentrations. As shown in Fig. 9c, both yeast transformants, one with the empty vector (pDR196) and one expressing CsHAK7, could grow on high K^+^ (10 mM) medium. However, at the low K^+^ concentration (0.1 mM), the growth of the pDR196 yeast transformant was completely suppressed, while the growth of the CsHAK7 yeast transformant was rescued (Fig. 9c). This result suggested CsHAK7 is a functional K^+^ transporter.

## Discussion

Plant HAK/KUP/KT family is the largest gene family of K^+^ transporters, responsible for K^+^ acquisition and transport with the plant, especially in the case of insufficient K^+^ supply [20, 21]. Here, we identified 21 *CsHAK* genes in the tea plant [59, 60], analyzed their evolutionary relationships, gene structure, predicted protein motifs, cis-acting elements, expression patterns in different tissues and under various stresses. Importantly, we further functionally characterized CsHAK7 in K^+^-uptake yeast.

### Evolutionary conservation of the CsHAK family genes

Phylogenetic analysis showed that the HAK family genes are evolutionarily conserved in the tea plant. For instance, 20 conserved motifs were identified and evenly distributed in all CsHAK proteins sequences. Notwithstanding, several motifs specific belonging to a particular cluster of CsHAKs were observed (Fig. 2b, Table S4), which could contribute to the CsHAKs divergence (Table S4). The 21 CsHAKs classification into four subgroups (Fig. 1) was consistent with that of poplar, grape rice and wheat [38, 63].

### Expression patterns of *CsHAKs* are generally tissue-specific in tea plants

The location and pattern of gene expression probably reflect its function in a plant. On the whole, the expression patterns of *CsHAKs* are tissue-specific. Notably, several *CsHAKs* were expressed especially highly in roots, such as *CsHAK4* (cluster I), *CsHAK5* (cluster I), *CsHAK19* (cluster IV) (Fig. 4), suggesting that these genes facilitate K^+^ uptake from soil. The *CsHAK16* from cluster IV was exclusively and highly expressed in flowers while in other tissues its expression levels were extremely low, suggesting that it plays a critical role in the tea plant reproductive tissue.

The number of plant *HAK/KUP/KT* genes in clusters II and III is much larger than in clusters I and IV. This was widely observed in various plant species as well as in tea plants (Fig. 4) [38]. The expression of *HAK/KUP/KT* genes from clusters II and III across different tissues may explain why the *HAK/KUP/KT* genes play multiple functions in plants [18]. Some plant *HAK/KUP/KT* genes from clusters II and III in plants other than the tea plant not only mediated K^+^ uptake and transport at both low and high K^+^ supply, but also participated in plant growth and development. For example, knockout of *AtKUP4* impaired root hair elongation [29] and mutation of *AtKT2/KUP2* caused reduced hypocotyl length in *Arabidopsis* [51]. Surprisingly, these phenotypes of mutants could not be rescued in the presence of high K^+^ concentration. In tea plants, these genes (*CsHAK1*, *2*, *3*, *7*, *8*, *10*, *12*, *13*, *17*, *18* and *21*) from cluster II and III were also highly expressed across eight tissues (Fig. 4), implying that they also play multiple roles in the tea plant growth and development.

### Potential role of *CsHAKs* in K^+^ deficiency and stress responses in tea plants

Transcriptional or post-transcriptional regulation K^+^ transporter genes are two major mechanisms underlying responses of plants exposed to K^+^-limitation conditions [19, 20, 69]. Studies of various plant species revealed induction of expression of *HAK/KUP/KT* genes by K^+^-starvation. For instance, in *Arabidopsis*, *AtHAK5* expression was markedly induced in roots under K^+^ limitation [28, 46]. In rice, the *OsHAK1* and *OsHAK5* were both greatly upregulated in roots after exposure to low K^+^ stress and they facilitated K^+^ translocation from root to shoot at low- or high- K^+^ concentrations [30, 32]. Recently, Qin et al. (2018) reported that the expression of *ZmHAK1* in maize roots increased appreciably in K^+^-deficiency conditions, while *ZmHAK5* expression was not changed [70]. ZmHAK1 together with ZmHAK5 played a crucial role in K^+^ uptake and translocation in maize after an exposure to K^+^ deficiency [70]. In rice, the majority of 17 *OsHAK* genes were upregulated by K^+^ starvation [71]. As expected, the expression of most tested *CsHAKs* was also induced after removal of K^+^, as assayed at the different time points, albeit to different levels (Fig. 5). It should be noted that the expression of the studied *CsHAKs* (CsHAK*8*, *12*, *18*, *20*) was induced after a short period of K^+^ starvation and then rapidly declined (Fig. 5), suggesting that these genes probably responded to the low K^+^ signal already at an early stage. The *CsHAK5*, *CsHAK19* and *CsHAK21* showed consecutive induction (Fig. 5), implying that these genes are involved in maintaining K^+^ homeostasis in tea plant under K^+^-deficient condition.

Previous studies reported that the HAK/KUP/KT genes were very responsive to various stress types and to plant hormones [35, 38, 72] and positively regulated stress responses in plants [66, 73]. Interestingly, many *cis*-acting elements related to plant growth and development, stresses and phytohormone responses were extensively distributed in the promoter regions of *CsHAKs* (Fig. 3), implying that *CsHAKs* also participate in stress and phytohormone responses. In rice, *OsHAK21* was significantly induced by salt stress and functioned in salt tolerance by maintaining the Na^+^/K^+^ homeostasis [33]. The *OsHAK5* transcript level also greatly increased in roots after exposure to salt stress and enhanced salt resistance of rice plants by elevating the ions ratio [K^+^]/[Na^+^] in shoots [30]. Most of the tested *CsHAKs* from clusters I-IV were upregulated by salt stress (Fig. 6). For instance, the expression level of *CsHAK7* rapidly increased at 6 h and reached a maximum at the time point of 24 h, and then decreased at 48 h time point (Fig. 6). *CsHAK1* transcript level increased continuously and stably during the first 24 h period and then quickly decreased at 48 h (Fig. 6), suggesting that *CsHAK1* responded to salt stress at an early stage. These results suggest the *CsHAK* genes have a role in enhancing salt resistance of the tea plant.

Drought stress was a major abiotic stress commonly threatens plant survival [67]. In rice, overexpression of *OsHAK1* improved tolerance to drought [34]. The drought tolerance of this transgenic rice was reportedly due to an enhancement of antioxidant enzymes and higher accumulation of a higher amount of proline [34]. We also found a few *CsHAKs* that were remarkably responsive to dehydration. The consistent upregulation of the transcript levels of *CsHAK1*, *8*, *21* expression across all time points (Fig. 7), suggested their involvement in drought stress responses in tea plants.

Similar to the reported effects of phytohormones on *HAK/KUP/KT* genes in other plants [35, 38], we found some *CsHAKs* were also responsive to phytohormones. This confirmed our expectations based the *cis*-elements of auxin and ABA responsiveness that we found in the promoter regions of several *CsHAKs* (Fig. 3). Indeed, there was a high correlation between the presence of the particular *cis*-elements in the gene’s promoter and this gene’s responsiveness to the particular phytohormone. For example: The *CsHAK11* transcript level was greatly induced in roots under IAA treatment (Fig. 8) while the promoter region of *CsHAK11* contained a *cis*-element of auxin responsiveness (Fig. 3). Also, the expression levels of *CsHAK7* and *CsHAK21* were rapidly upregulated under ABA treatment (Fig. S3) matching the presence of ABA-responsiveness *cis*-element(s) in the promoters. Interestingly, the upregulated expression of these genes in the roots of tea plant, echoes the impairment of ABA responses in Arabidopsis lateral root cells due to a mutation of AtKUP2/6/8 [29]. Incidentally, this Arabidopsis mutant also had enhanced cell expansion and impaired ABA responses in guard cells [29], all of which help link the effect of ABA on the *CsHAKs* expression to ABA regulation of K^+^ homeostasis in the whole tea plant.

### *CsHAK7* is a potentially key gene for K^+^ acquisition in the tea plant

Recently, the expression pattern and functional characterization of *AtHAK7* was investigated [52]. The results of this study showed that AtKUP7 was expressed in all tissues, including root, leaf, stem, flower, and silique, especially a higher expression level in stelar tissues and was shown to be involved in transport K^+^ into xylem under K^+^-limited conditions [52]. AtKUP7 shares almost highest amino acids identify with CsHAK7 in tea plant. Quantitative results showed *CsHAK7* also expressed in all tissues of tea plant, especially a relatively higher in mature leaves and main vein (Fig. 9), suggested a similarity in their function. Indeed, the rescue by *CsHAK7* of the K^+^-transport-disabled yeast mutant in a K^+^-deficient medium suggests that *CsHAK7* functions in a high-affinity K^+^ uptake, resembling the demonstrated involvement of AtKUP7 in K^+^ transport into xylem under K^+^-limitation [52]. Based on the relatively higher *CsHAK7* expression in mature leaves and main vein (Fig. 9), we speculate that *CsHAK7* plays a critical role in K^*+*^ transfer under K^+^-deficiency conditions from mature leaf to new shoots in the tea plant. This function could be perhaps related to the general mechanism underlying the known phenomenon of K^+^ mobilization from source leaves to young developing leaves temporarily overcoming K^+^ deficiency and delaying the appearance of its symptoms in the leaves [74, 75]. Based on the upregulation of CsHAK7 expression by salt and drought stresses, ABA treatment, and by K^+^ deficiency, we speculate that CsHAK7 fine-tunes K^+^ fluxes across the tea plant plasma membrane to adjust the turgor pressure as a part of the plant’s stress tolerance. Moreover, *CsHAK7* expression was also induced by IAA, suggested a role of CsHAK7 in tea plant growth. Other functions of CsHAK7 and its physiological role in the tea plant warrant future in-depth exploration.

## Conclusions

In conclusion, a total of 21 CsHAK family members were identified in tea plants. Based on the phylogenetic and structural features analysis, all 21 *CsHAKs* were classified into four clusters (I-IV). *cis*-acting elements related to plant growth and development, stresses and plant hormone were found in the *CsHAKs* promoter regions. The analysis of issue-specific and various stress types-induced expression patterns suggested that *CsHAKs* function in K^+^ uptake and stress responses in the tea plant root. Importantly, we demonstrated CsHAK7 participation in K^+^ uptake in yeast. Taken together, our findings offer a good platform for further characterization of the multiple physiological roles of CsHAKs in the tea plant.

## Methods

### Identification *CsHAK*s in tea plants

To identify and annotate *CsHAKs* in tea plants, both the Hidden Markov Model (HMMER) profile and local BLAST searches were combined to analyze the genomic data. Firstly, the HMM profile (PF02705) [76] of the CsHAK proteins conserved domain was used to screen protein sequences from tea plant (*Camellia sinensis* vs ‘Shuchazao’) genome [59]. Secondly, 13 *Arabidopsis* AtKUP/HAK/KT protein sequences and 27 rice OsHAK protein sequences were used (as described previously for *Arabidopsis* [28] and for rice [38]) as queries to screen against the tea plant genome database with BLASTP program (e-value <1e^− 5^). Finally, the results of the two methods were merged to obtain candidate *CsHAK* family members and further verified for the presence of complete *CsHAK* domains by screening against the CCD (https://www.ncbi.nlm.nih.gov/cdd/), InterProScan [77] and SMART [78] databases. The physical and chemical parameters of the CsHAK proteins, i.e., their molecular weights and isoelectric points were calculated by the ExPasy website (https://web.expasy.org/protparam/). The subcellular localization of the CsHAK proteins was predicted using WoLFPSORT (http://www.genscript.com/psort/wolf_ psort.html). The amino acid lengths of the CsHAK proteins were obtained from the genomic website [59, 60]. Their transmembrane domains were predicted by TMHMM2 program (www. cbs.dtu.dk/services/TMHMM/).

### Phylogenetic tree construction of CsHAKs

The protein sequences of HAKs in other plant species were retrieved from the NCBI database (https://www.ncbi.nlm.nih.gov/) and were used to perform multiple sequence alignments by ClustalW program (Version 2.1; http://www.clustal.org/). Phylogenetic trees were constructed based on the protein sequences of 92 HAKs using the neighbor-joining method of the program MEGA6.0 with bootstrap (1000 replicates) analysis [79]. Moreover, another phylogenetic tree was also constructed using 21 protein sequences of CsHAKs for further analysis. The genes loci of *HAKs* in other plant species are listed in Table S2.

### Analysis of gene structure, motifs distribution and *cis*-acting elements of the *CsHAKs*

The gene structure display server (GSDS) 2.0 program (http://gsds.cbi.pku.edu.cn/) was used to analyze the *CsHAKs* gene structure. The conserved motifs of the CsHAK proteins were predicted using MEME program (http://meme.nbcr.net/meme/cgi-bin/meme.cgi) by submitting the predicted protein sequences. The parameters of MEME were used as follows: maximum number of motifs, 20; minimum motif width, 6; and maximum motif width, 70. In addition, the promoter sequences of 2000 bp upstream of the transcription start sites of each *CsHAKs* were retrieved from tea plant genome website [60], and the 2000 bp promoter regions of *CsHAKs* were analyzed in the PlantCARE program (http://bioinformatics.psb.ugent.be/webtools/plant care/html/).

### Expression profiles of *CsHAKs* detected by transcriptome data

For tissue-specific expression of *CsHAKs* analysis, the raw transcriptome sequencing data (SRA accession no. SRP056466) [59] from eight tea plant tissues (including root, stem, apical bud, young leaf, mature leaf, old leaf, flower and fruit) were downloaded from the Sequence Read Archive (SRA) database (https://www.ncbi.nlm.nih.gov/sra/). Next, we mapped the raw transcriptome sequencing data to the tea plant genome and calculated the FPKM value according to the previous methods [59], and then the expression levels of *CsHAKs* were visualized by the “pheatmap” package implemented in R (https://cran.r-project.org/web/packages/pheatmap/index.html).

### Plant material and growth conditions

Two-year-old tea cutting seedings (*Camellia sinensis* L. cv. ‘shuchazao’) were obtained from Dechang Tea Fabrication Base at Shucheng County (Anhui province, China). Tea seedlings with uniform size were used for hydroponic culture. Tea seedlings were grown in full basal nutrient solution in a growth chamber at state Key Laboratory of Tea Plant Biology and Utilization, Anhui Agricultural University (Hefei, China) for 1 month to produce well developed roots. The growth condition of tea plants was controlled as following: light intensity of 200 μmol phtotons m^− 2^ s^− 1^ for 14 h per day, day/light temperature of 25/22 °C and relative humidity of 70%. The composition of the basal nutrient solution was as reported previously [80]. The pH of the nutrient solution was adjusted to 4.5, and it was replaced once a week.

### Plant treatments

For K^+^ depletion experiments, tea cutting seedlings were grown in a solution containing 0.513 mM K^+^ for 1 month and then transferred into a treatment solution in which 0.2065 mM K_2_SO_4_ and/or 0.1 mM KH_2_PO_4_ was replaced by 0.2065 mM Na_2_SO_4_ and/or 0.1 mM NaH_2_PO_4_. For salinity stress experiments, the treatment solution consisted of the full basal nutrient solution supplemented with 200 mM NaCl; the EC of the treatment solution was 17.77 ms/cm and the control is 0.465 ms/cm; for drought stress experiment, the full basal nutrient solution contained 20% (g/v) polyethylene glycol 6000 (PEG 6000) [68]. For experiments with phytohormones, the treatment solution was the full basal nutrient solution with added 100 μM ABA or 10 μM IAA [81]. After treatments, tissue samples were collected at different time points, 1 h, 6 h, 12 h, 24 h and 48 h, and were immediately frozen in liquid nitrogen and stored at − 80 °C for further analysis.

For tissue-specific expression analysis, we collected different tea plant tissues: leaf, root, stem, apical bud, MV (major vein) and VB (vascular bundle, peeled from the stem), as described previously [58].

### Gene expression analysis using qRT-PCR

Total RNAs from the various sampled tissues were isolated using the modified CTAB method reported previously [80]. First-strand cDNAs were synthesized using the PrimeScript RT Reagent Kit with gDNA Eraser (Takara, Dalian, China) according to the manufacturer’s protocol. qRT-PCR was run on a Bio-Rad CFX96 machine with SYBR® Premixm Ex Taq™ (TaKaRa, Japan). *CsGAPDH* (TEA025584.1) was used as an internal control. Data were analyzed with Opticon monitor software (Bio-Rad). All primers for qRT-PCR were designed using Primer 5.0 software and primer sequences are listed in Table S1. All the experiments were performed with three biological replicates. The 2^–ΔCT^ method was used to calculate the relative expression level [82, 83].

### Subcellular localization analysis of CsHAK7

The full-length coding sequence of *CsHAK7* without the stop codon was amplified by RT-PCR using primers (Table S1) containing double restriction sites of SpeI and BamHI. The purified PCR products were digested with SpeI and BamHI, and then fused to the pCAMBIA1305.1:GFP expression vector to generate a fusion protein CsHAK7::GFP, driven by the CaMV 35S promoter. After validation by sequencing, the plasmids of both CsHAK7::GFP and AtPIP2A::mCherry (Plasma membrane (PM) marker) [68] or both 35S:GFP (as a control) and 35S:AtPIP2A::mCherry, were co-transformed into the *Agrobacterium tumefaciens* strain EHA105. The bacterial cells were collected by centrifugation and resuspended in a solution (pH 5.7) containing 10 mM MES, 10 mM MgCl_2_, and 200 mM acetosyringone (AS). Next, a *Nicotiana benthamiana* plant with four to five leaves (approximately 1 month old) was used for Agrobacterium transient transformation experiment. Cell suspensions at an optical density of 0.3 (at 600 nm) were infiltrated into the leaves of *Nicotiana benthamiana* using a needle-free syringe. 48 or 72 h after infiltration, we examined the tobacco epidermis cells by confocal laser scanning microscope (LSM410; Carl Zeiss); the green GFP fluorescence was elicited by excitation at 488 nm and observed via a 535 nm filter, and the red fluorescence of mCherry was elicited by excitation at 543 nm and observed via a 585 nm filter, as previously described [84].

### Yeast complementation assay

To elucidate the function of CsHAK7 in K^+^ uptake in yeast, the coding sequence of *CsHAK7* was amplified with RT-PCR using primers (Table S1), and then the purified PCR products were inserted into the yeast expression vector pDR196 under the control of the inducible PMA promoter [85]. The plasmids of the expression vectors (pDR196 and pDR196-CsHAK7) were transformed into the R5421 strain, a K^+^-uptake-defective strain of *Saccharomyces cerevisiae* [30]. The yeast cell transformation method was employed as described previously [84]. Arginine phosphate (AP) medium was used for subsequent growth assays that were carried out as described previously [30, 62]. The yeast complementation tests were performed on solid AP medium, and the plates were incubated at 30 °C in dark condition for 3–5 d.

## Supplementary information

**Additional file 1: Fig. S1.** The predicted transmembrane helices of the CsHAKs. The transmembrane domains of CsHAKs proteins were predicted using an internet server, TMHMM2(www.cbs.dtu.dk /services/TMHMM/), and the red peaks represent the predicted transmembrane regions of proteins.

**Additional file 2: Fig. S2.** *Cis*-elements in promoters of CsHAKs in tea plants.

**Additional file 3: Fig. S3.** Expression profiles of *CsHAKs* in the tea plant root in response to ABA treatment. The mean value was calculated from three independent replicates. Error bars indicate standard errors of three biological replicates. Significant differences from the control in each group are indicated by different letters (*P* < 0.05, one-way ANOVA). ns, no siginificance.

**Additional file 4: Table S1.** List of primers used in this study.

**Additional file 5: Table S2.** HAK/KUP/KT family genes in *Arabidopsis thaliana*, *Oryza sativa*, *Populus trichocarpa*, *Vitis vinifera* .

**Additional file 6: Table S3.** Information of the conserved motifs in CsHAKs.

**Additional file 7: Table S4.** Conserved motifs identified from the *CsHAK*s in tea plants.

**Additional file 8: Table S5.** *Cis*-acting elements identified from the *CsHAKs* promoters of tea plants.

**Additional file 9: Table S6.** Expression levels of *CsHAKs* in different tissues of tea plants.

## Data Availability

All data generated or analyzed during this study were included in this published article and the additional files.

## Abbreviations

HAK/KUP/KT: (High-affinity K^+^ transporter / K uptake permease / K^+^ transporter)
TM: Transmembrane
GFP: Green fluorescent protein
RFP: Red fluorescent protein
MW: Molecular weight
pI: Isoelectric points
TAIR: The *arabidopsis* information resource
GSDS: Gene structure display server
RNA-seq: RNA sequencing
FPKM: Fragments per kilobase million
PM: Plasma membrane
ABA: Abscisic acid
IAA: Indole-3-acetic acid
NAA: 1-naphthylacetic acid
PEG 6000: Polyethylene glycol 6000
SRA: Sequence read archive
MV: Major vein
VB: Vascular bundle
AP: Arginine phosphate.
