# Supplementary figures and images for: HAK/KUP/KT family potassium transporter genes are involved in potassium deficiency and stress responses in tea plants (Camellia sinensis L.): expression and functional analysis
Source: BMC Genomics. 2020 Aug 13;21:556. doi: 10.1186/s12864-020-06948-6 (PMC7430841; doi:10.1186/s12864-020-06948-6)

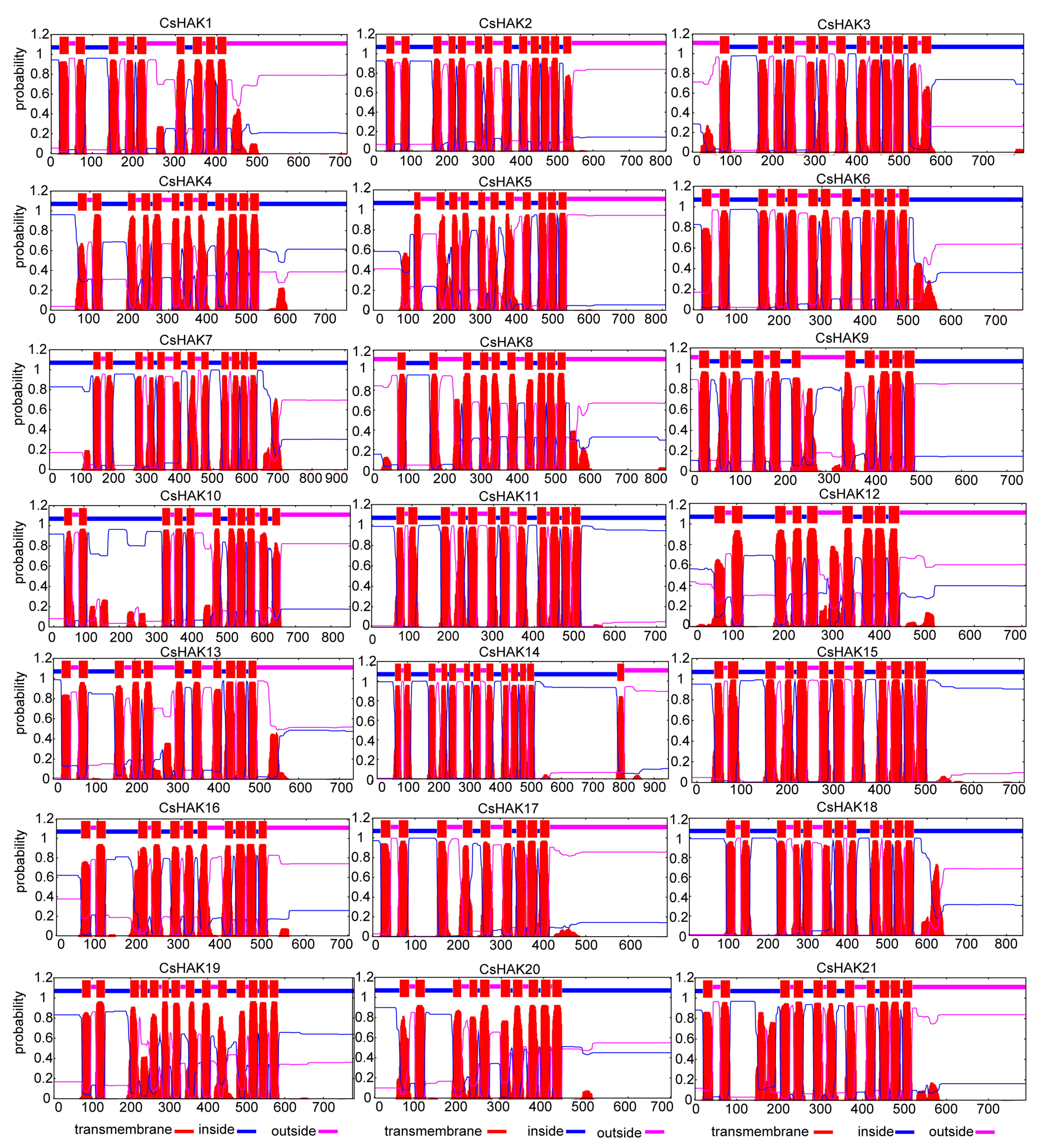

Supplement: Supplementary file 1 — Additional file 1: Fig. S1. The predicted transmembrane helices of the CsHAKs. The transmembrane domains of CsHAKs proteins were predicted using an internet server, TMHMM2(www.cbs.dtu.dk /services/TMHMM/), and the red peaks represent the predicted transmembrane regions of proteins. [file 12864_2020_6948_MOESM1_ESM.tif]

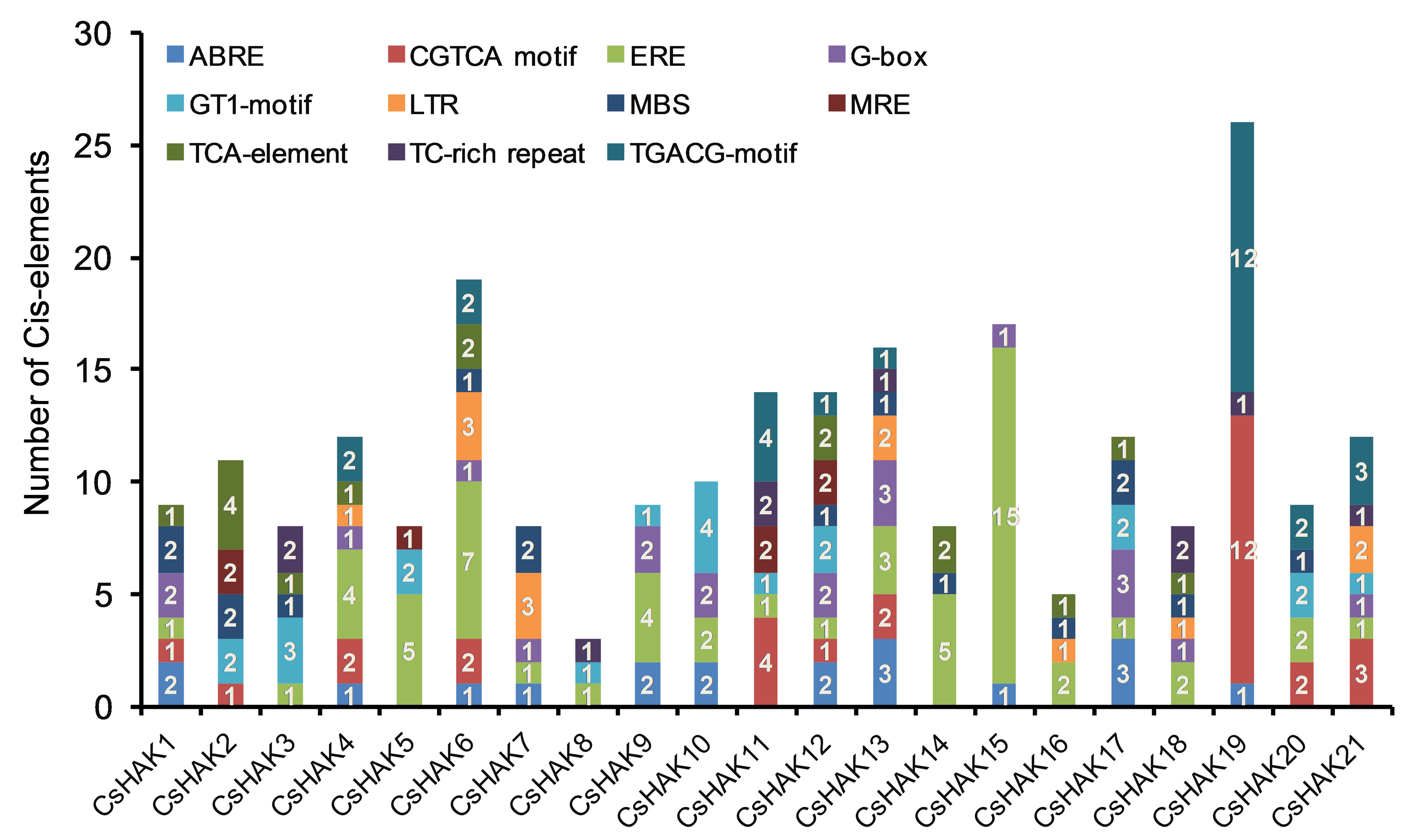

Supplement: Supplementary file 2 — Additional file 2: Fig. S2. Cis-elements in promoters of CsHAKs in tea plants. [file 12864_2020_6948_MOESM2_ESM.tif]

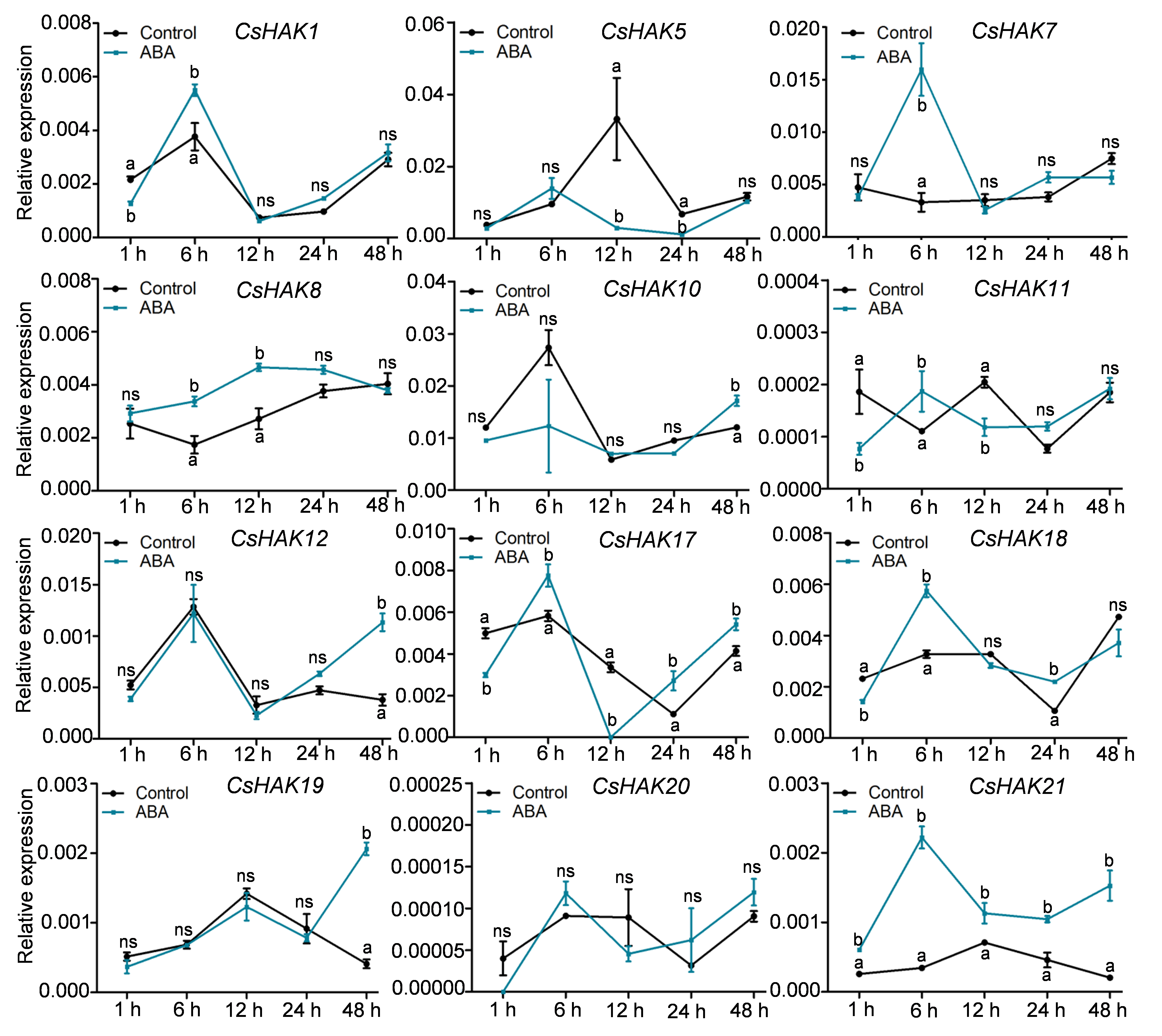

Supplement: Supplementary file 3 — Additional file 3: Fig. S3. Expression profiles of CsHAKs in the tea plant root in response to ABA treatment. The mean value was calculated from three independent replicates. Error bars indicate standard errors of three biological replicates. Significant differences from the control in each group are indicated by different letters (P < 0.05, one-way ANOVA). ns, no siginificance. [file 12864_2020_6948_MOESM3_ESM.tif]
