# Supplementary material for: HAK/KUP/KT family potassium transporter genes are involved in potassium deficiency and stress responses in tea plants (Camellia sinensis L.): expression and functional analysis
Source: BMC Genomics. 2020 Aug 13;21:556. doi: 10.1186/s12864-020-06948-6 (PMC7430841; doi:10.1186/s12864-020-06948-6)
Supplement: Supplementary file 7 — Additional file 7: Table S4. Conserved motifs identified from the CsHAKs in tea plants. [file 12864_2020_6948_MOESM7_ESM.docx]

**Table S4**. Conserved motifs identified from the *CsHAK*s in tea plants*.*

| Motif1 | 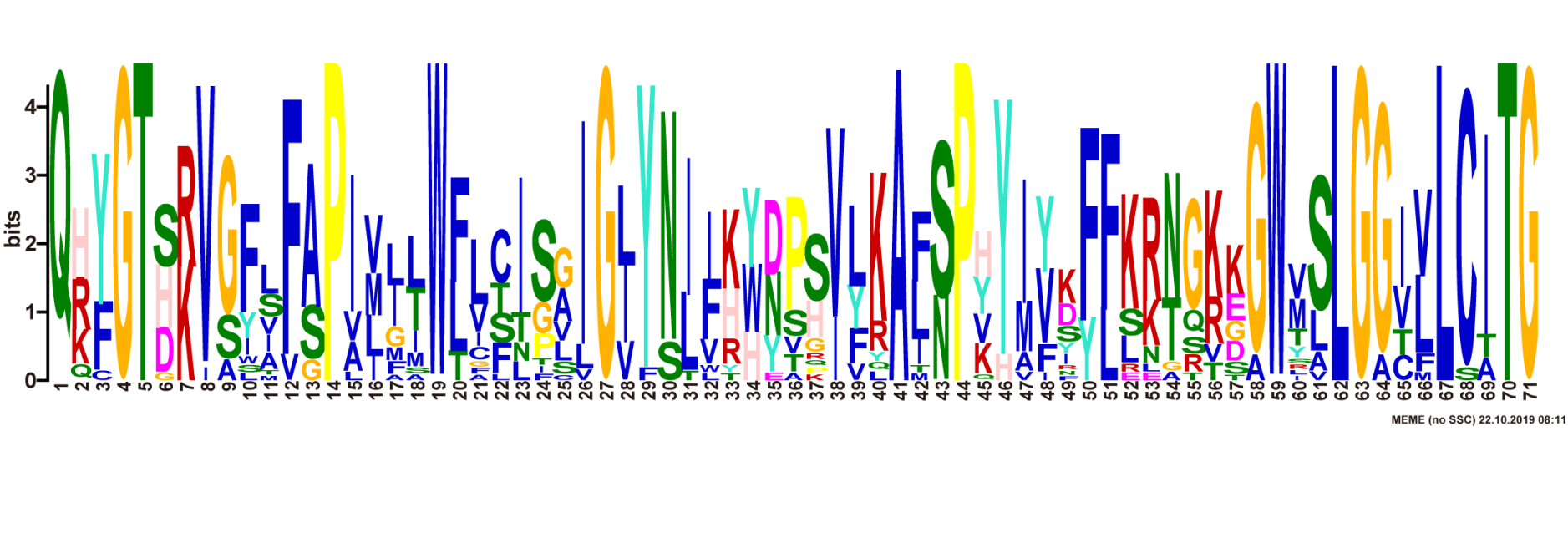   \|  \| \| --- \| |
| --- | --- | --- |
|  | QHYGTSKVGFLFAPIVJLWFLCISGIGIYNJIKYBPSVLKAFSPHYIYKFFKRNGKKGWVSLGGIVLCITG |
| Motif2 | 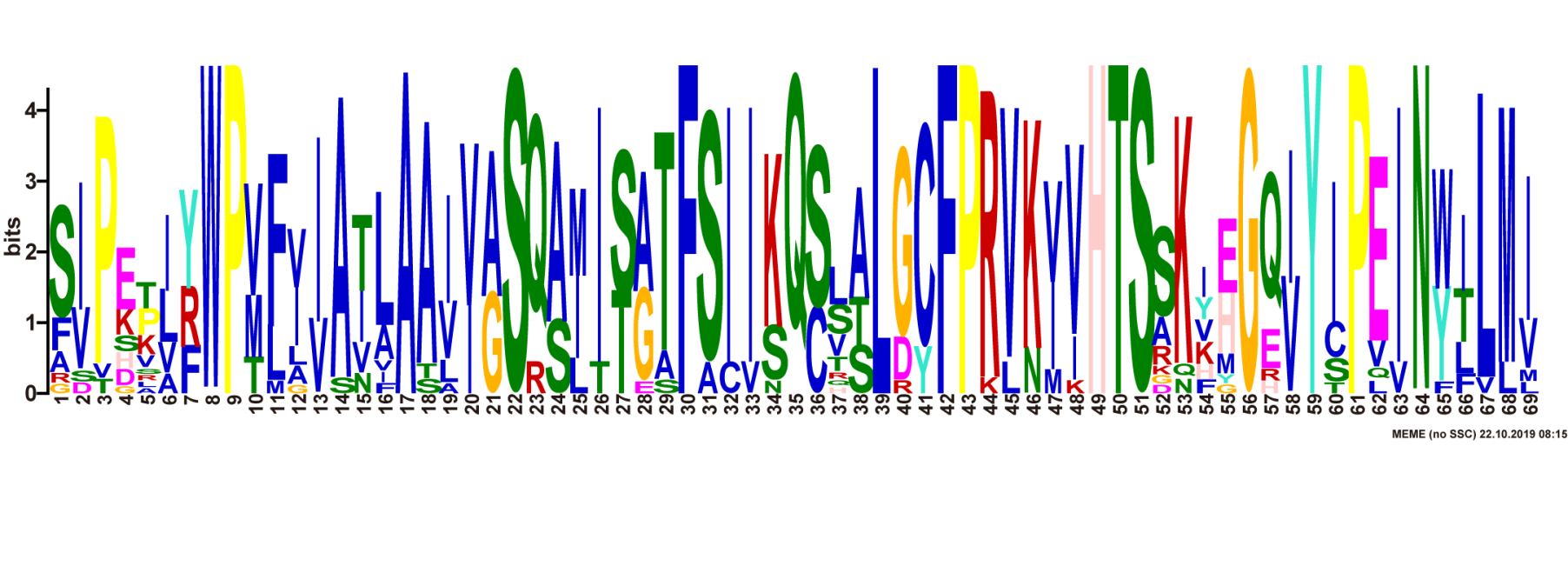 |
|  | SIPETJYWPVFIIATLAAIVASQAIISATFSIIKQSLALGCFPRVKVVHTSSKIEGQIYIPEINWILMI |
| Motif3 | 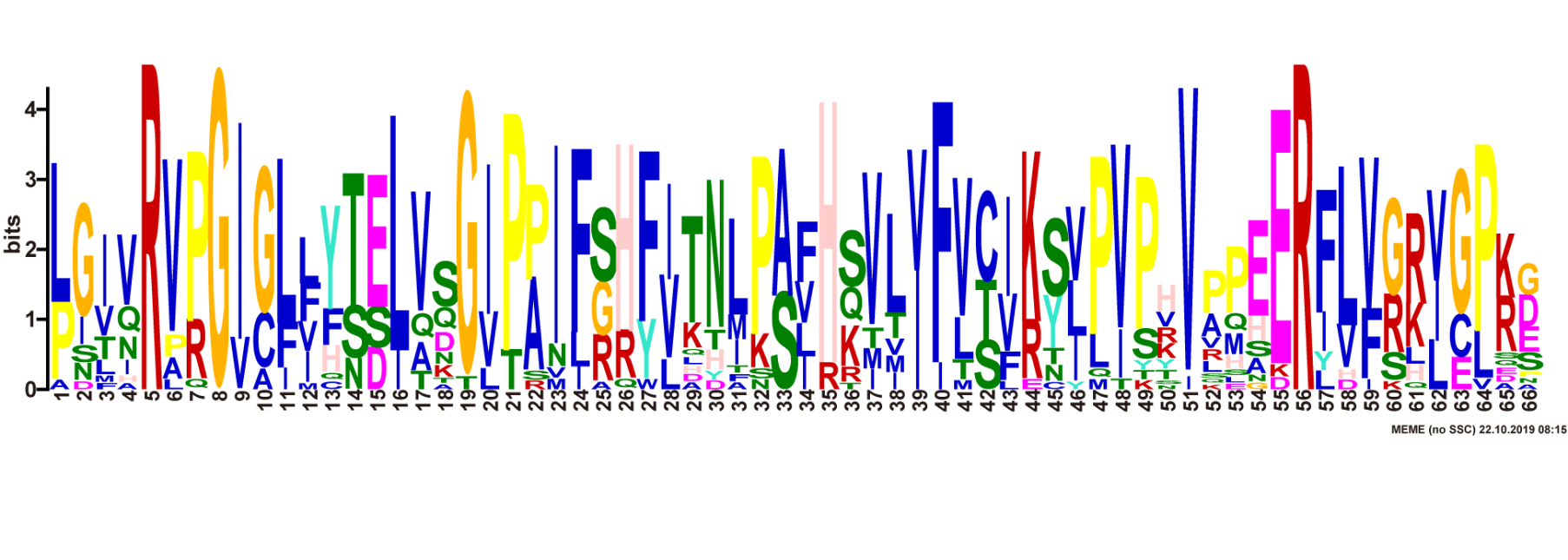 |
|  | LGIVRVPGIGLLYTELVSGIPPIFSHFITNLPAFHSVLVFVCIKSVPVPHVPPEERFLVGRVGPKD |
| Motif4 | 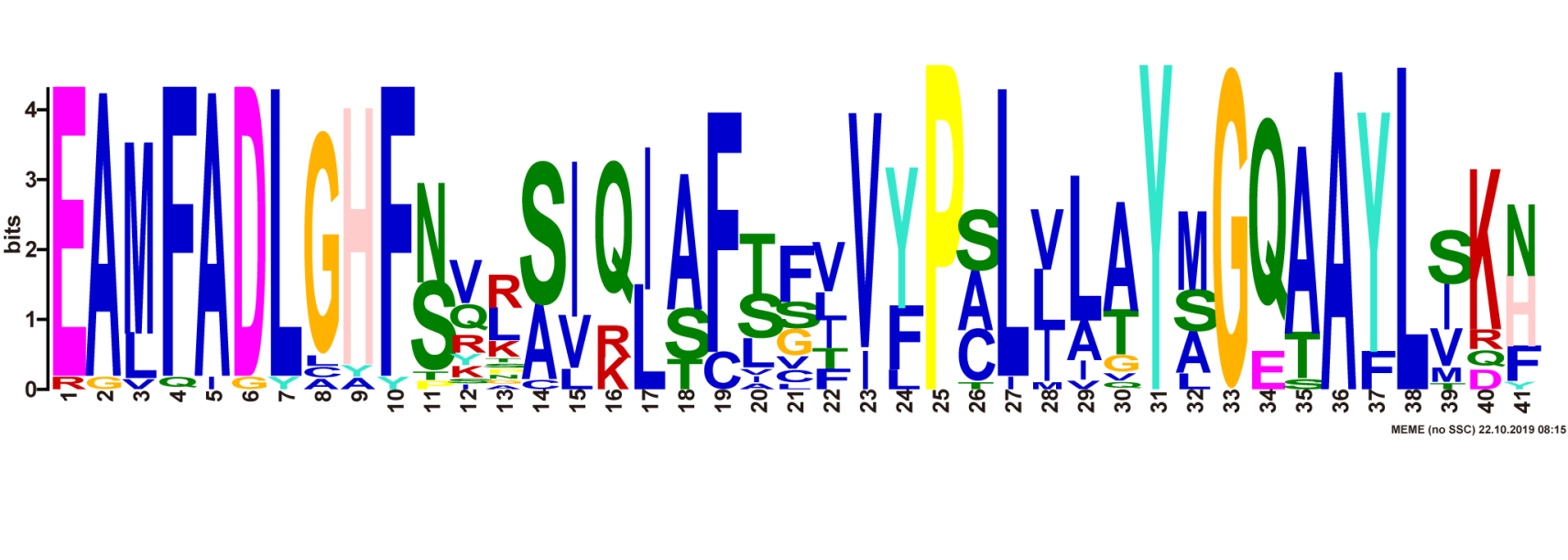 |
|  | EAMFADLGHFNVRSIQJAFTFVVYPALLLAYMGQAAYLSKH |
| Motif5 | 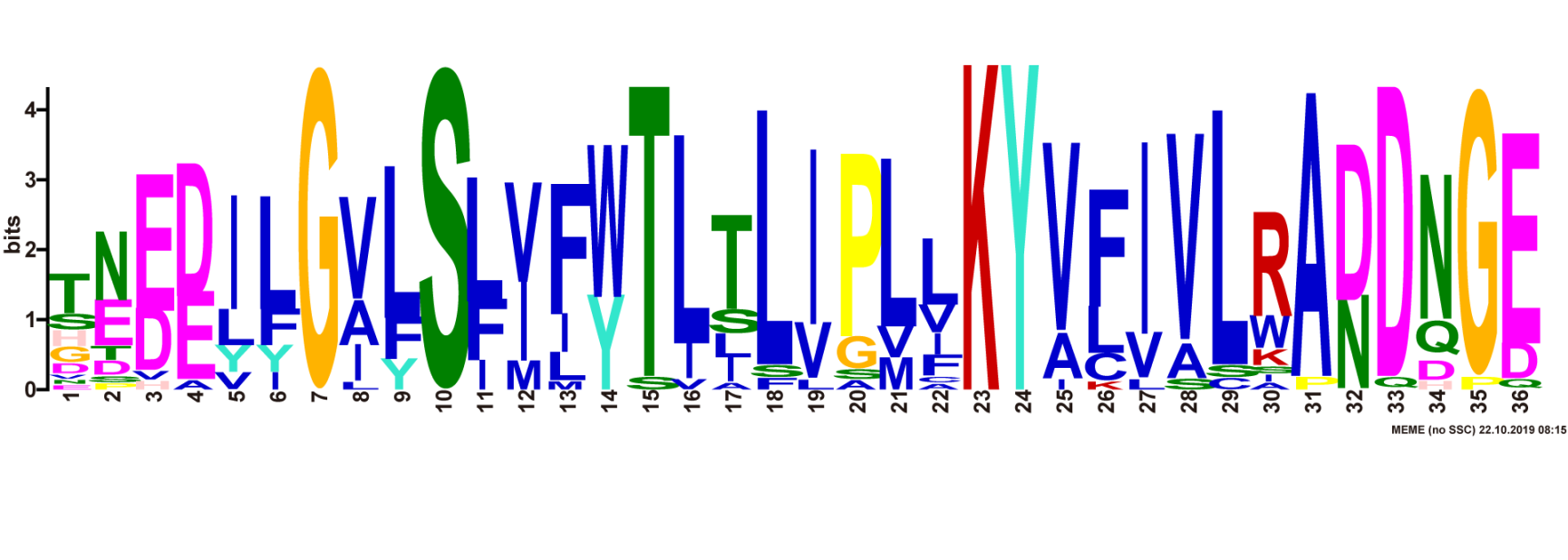 |
|  | TNEDILGVLSLVFWTLTLIPLLKYVFIVLRABDNGE |
| Motif6 | 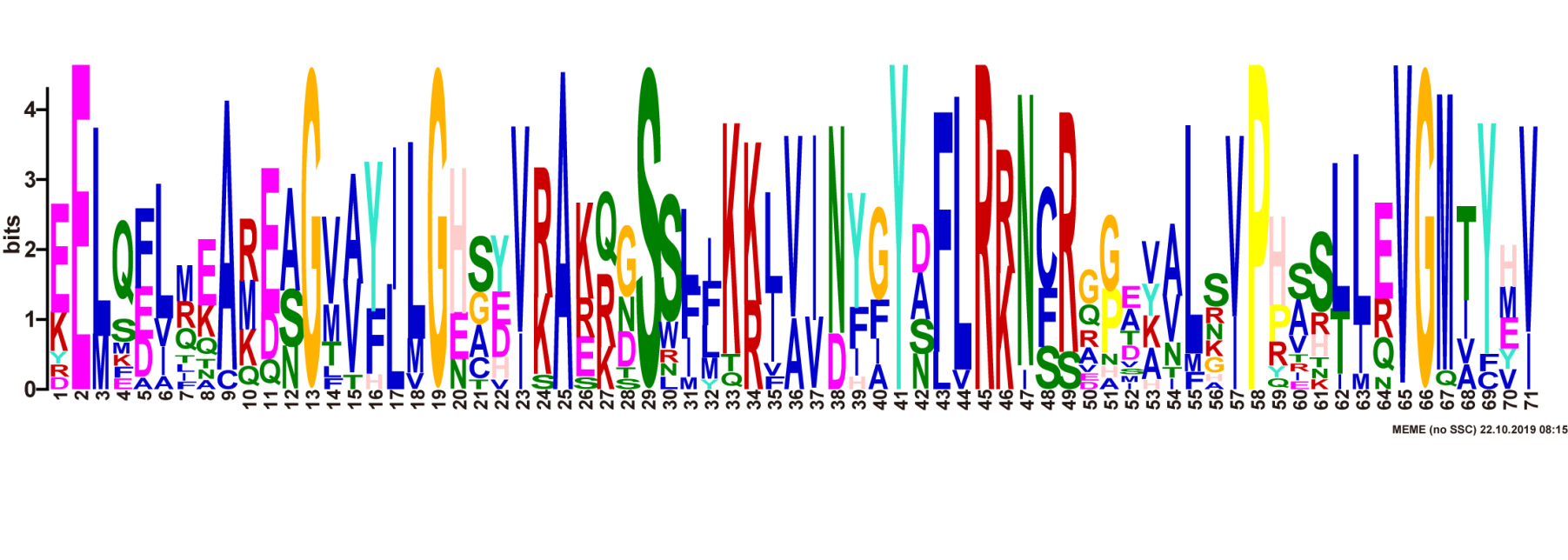 |
|  | EELQFLMEAREAGVAYJLGHSYVRAKQGSSLIKKJVINYGYDFLRKNCRGGEVALSVPHASLLEVGMTYHV |
| Motif7 | 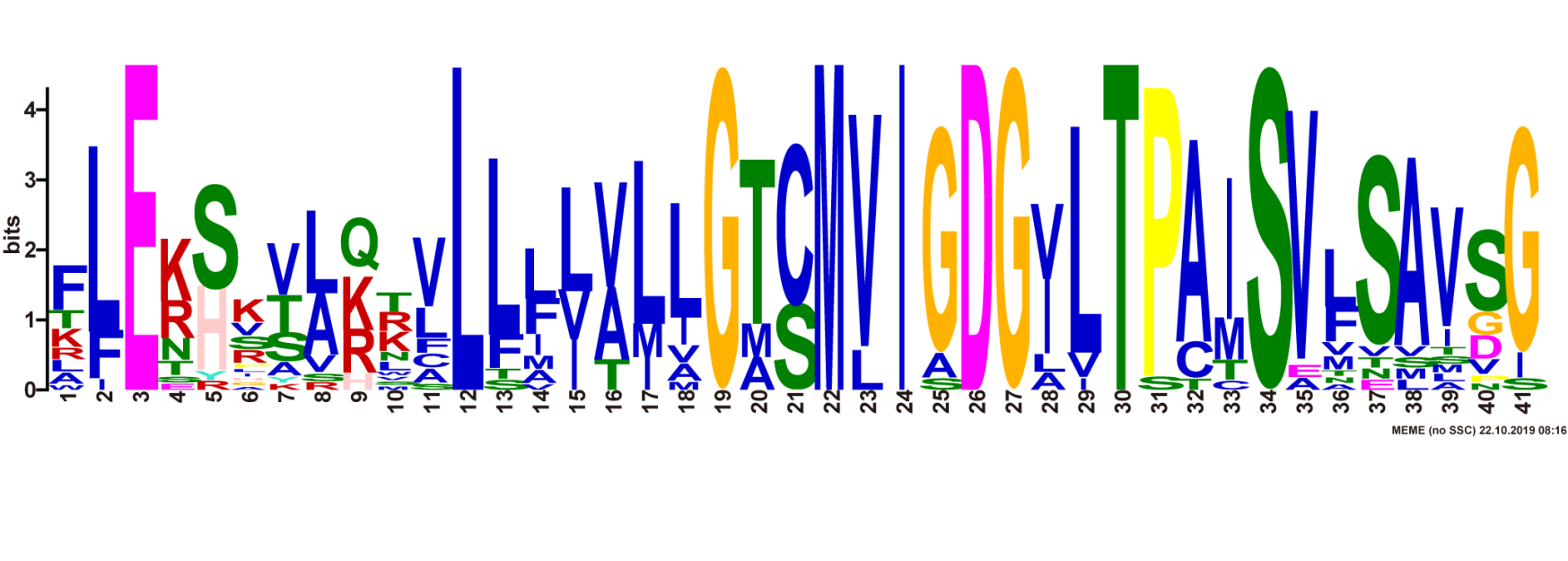 |
|  | FLEKSKVLQKVLLLLVLLGTCMVIGDGILTPAISVLSAVSG |
| Motif8 | 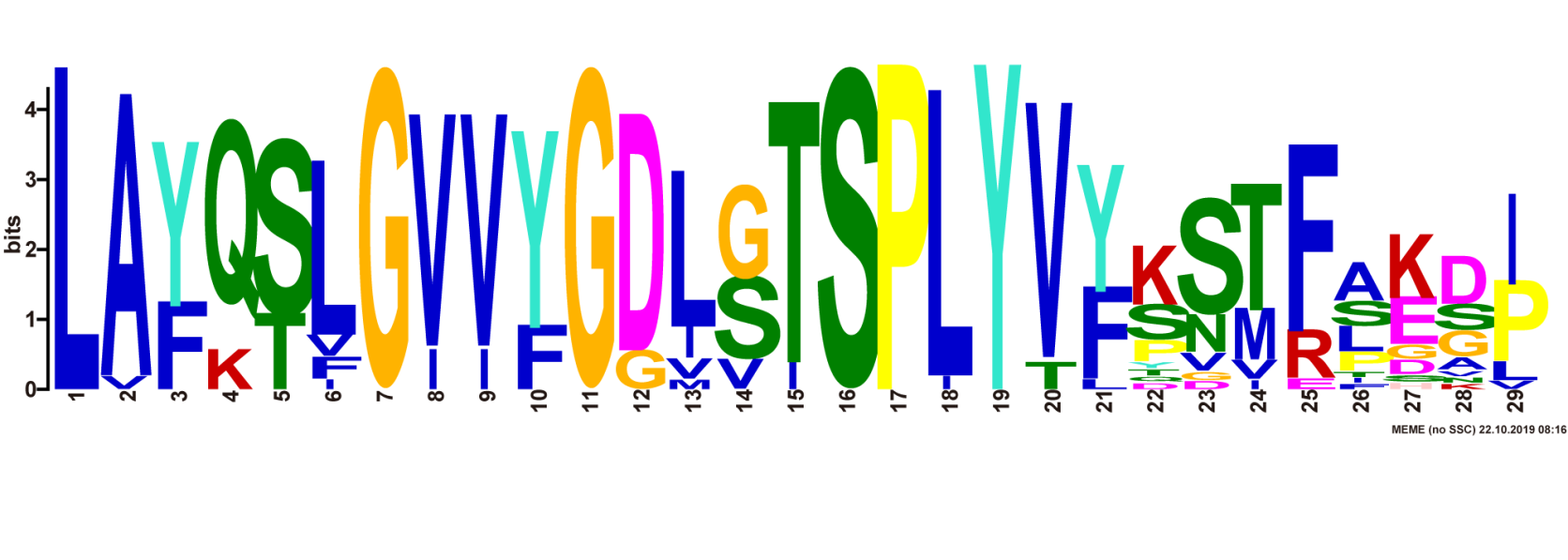 |
|  | LAYQSLGVVYGDLGTSPLYVYKSTFAKDI |
| Motif9 | 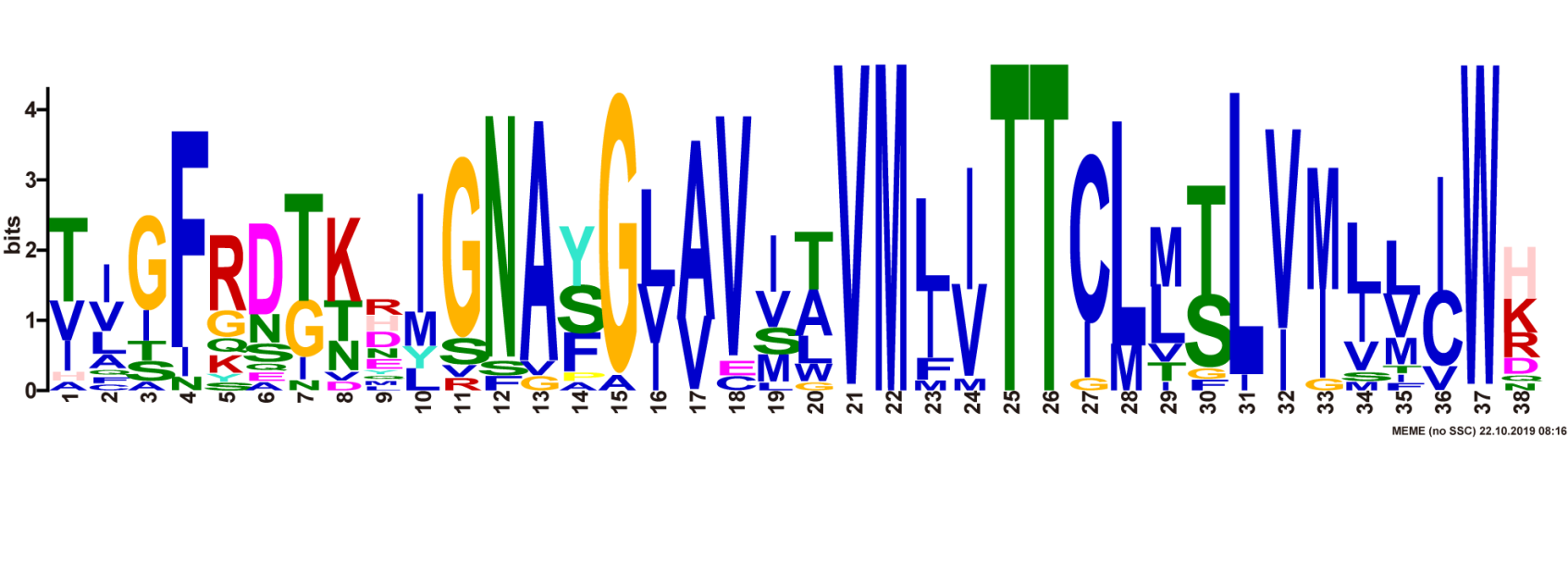 |
|  | TIGFRDTKRIGNAYGLAVITVMJITTCLLTLVMJLIWH |
| Motif10 | 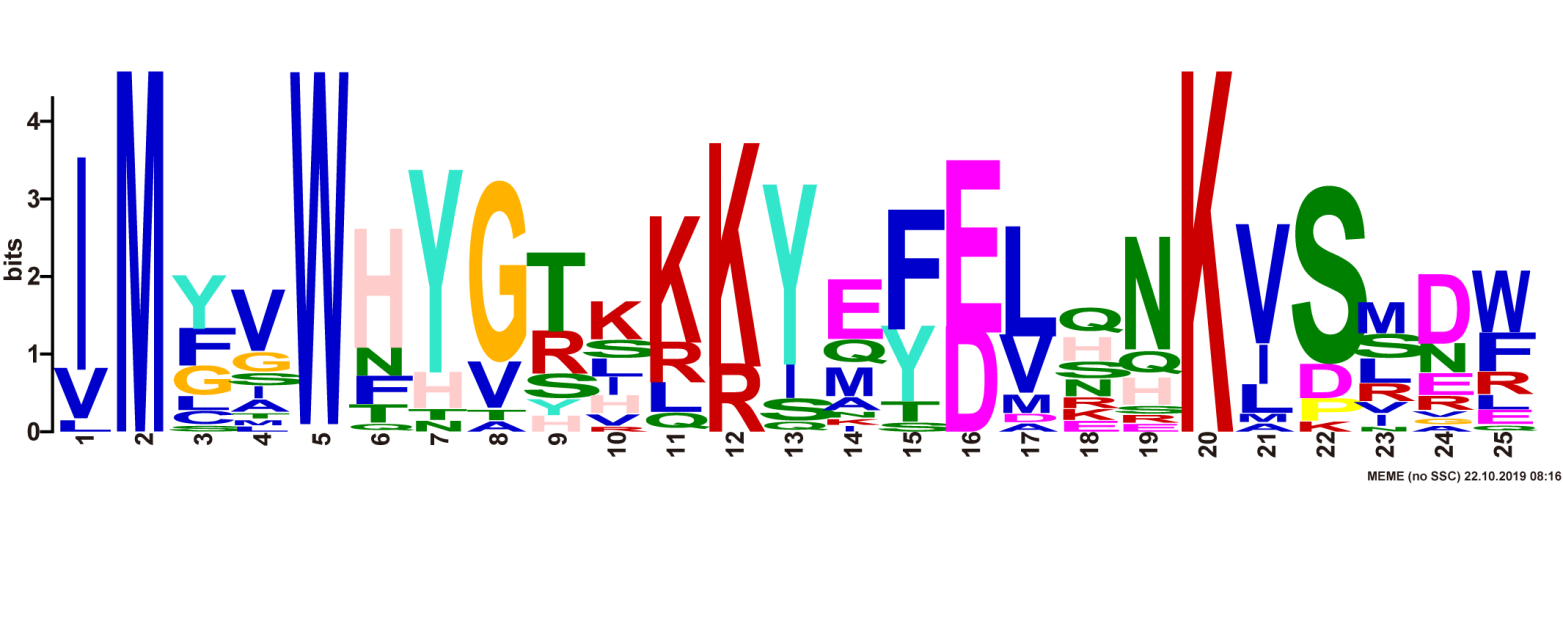 |
|  | IMYVWHYGTKKKYEFELQNKVSMDW |
| Motif11 | 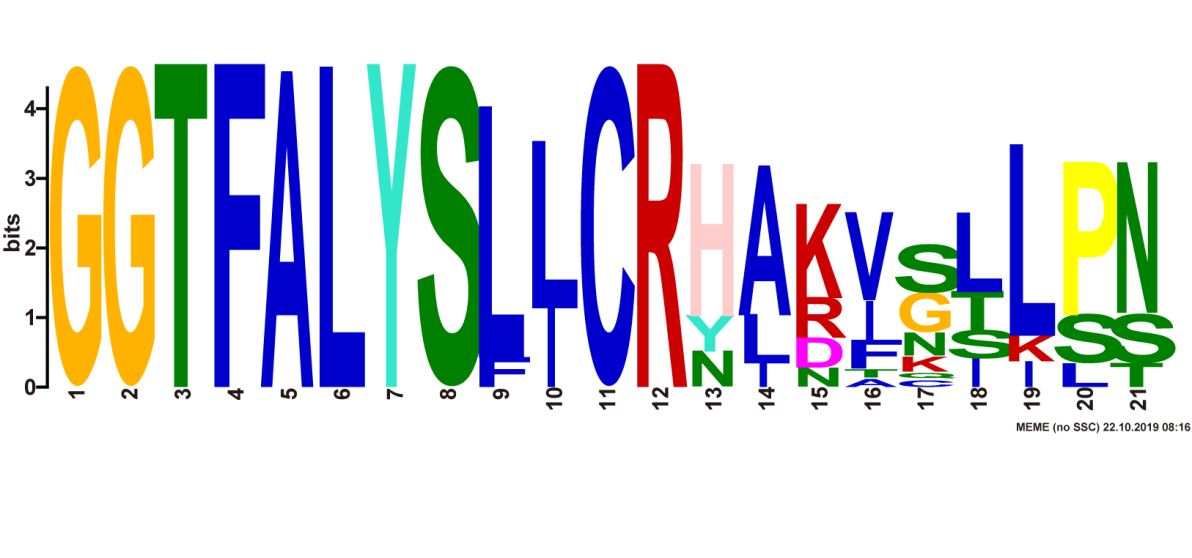 |
|  | GGTFALYSLLCRHAKVSLLPN |
| Motif12 | 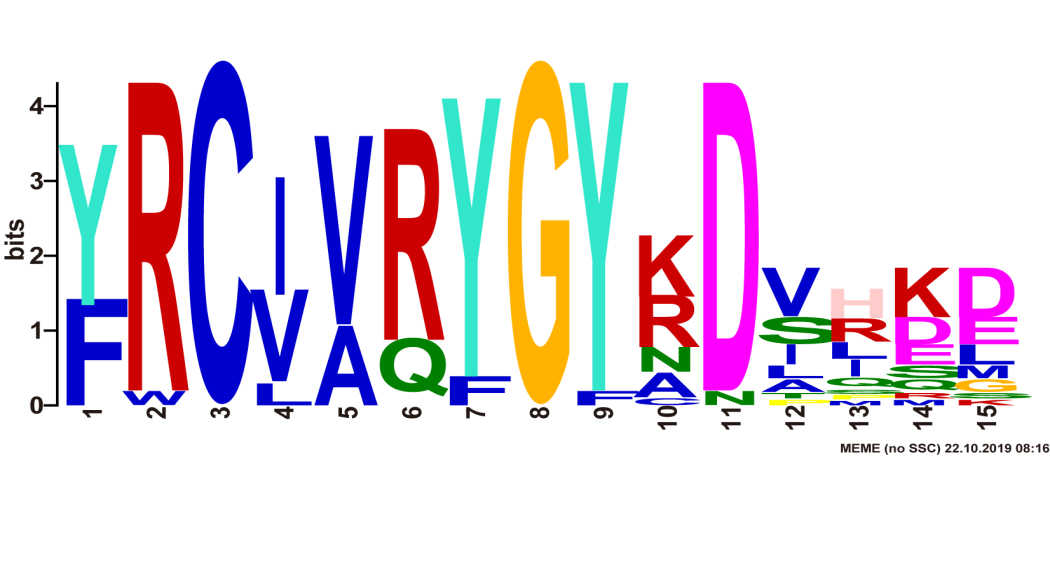 |
|  | YRCIVRYGYKDVHKD |
| Motif13 | 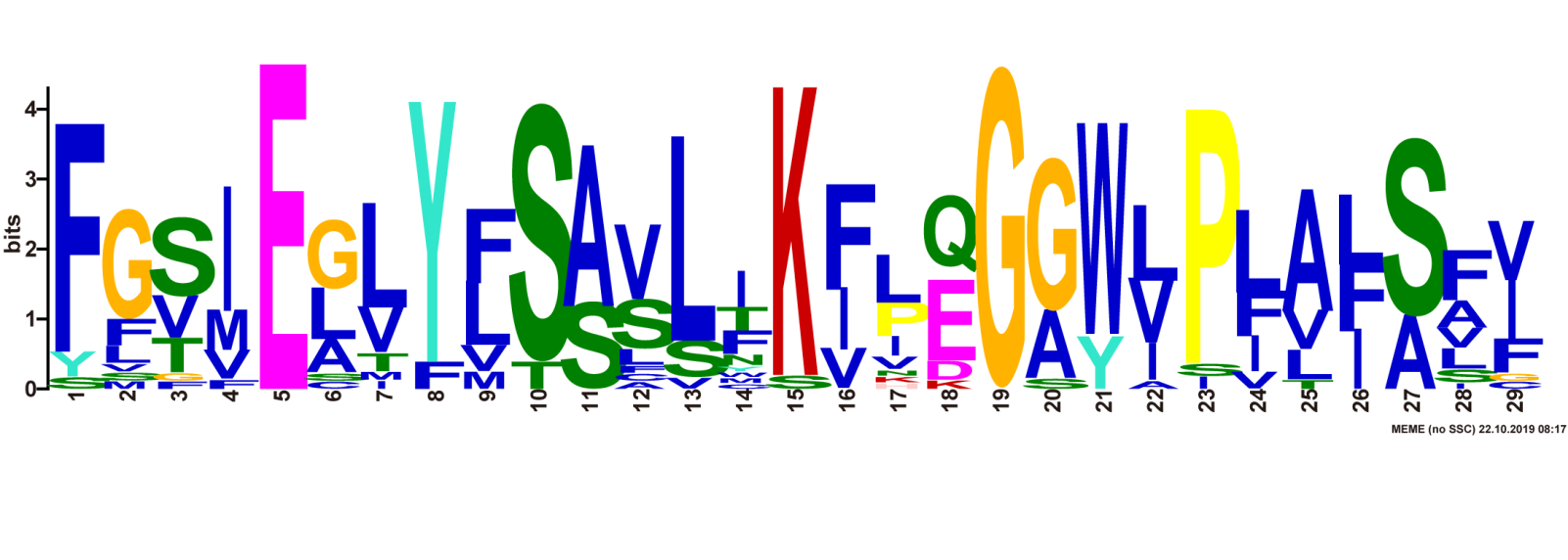 |
|  | FGSIEGLYFSAVLIKFLZGGWLPLALSFV |
| Motif14 | 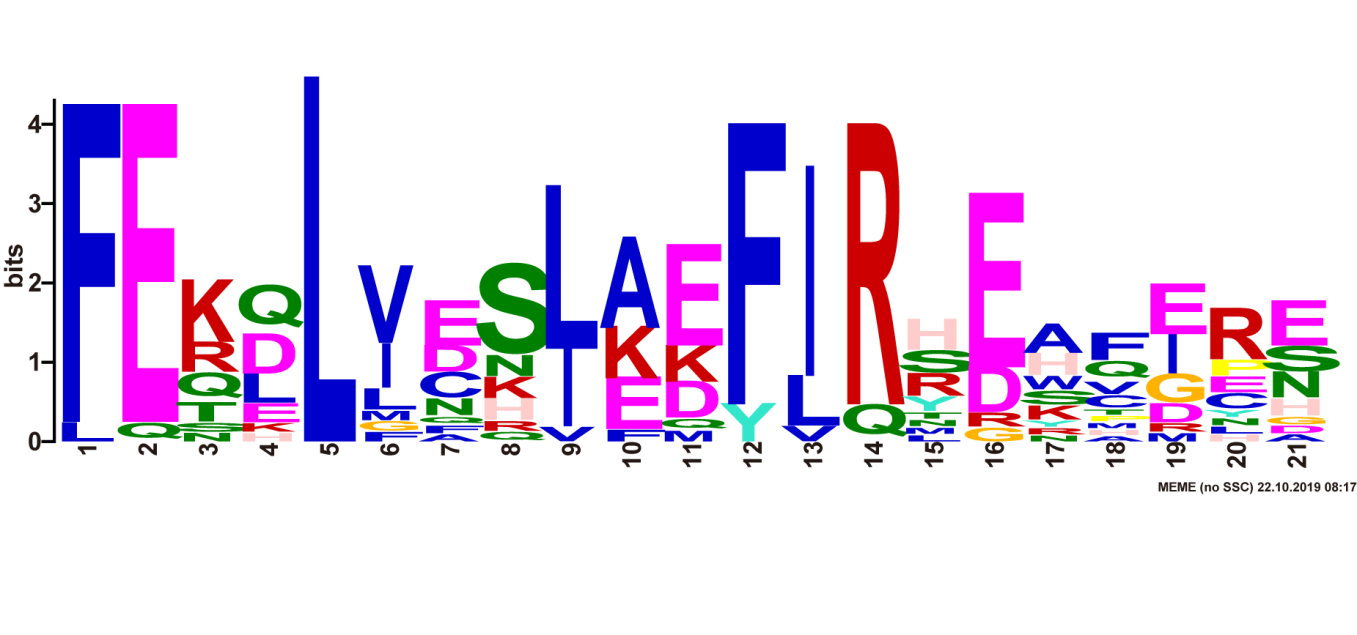 |
|  | FEKQLVESLAEFIRHEAFERE |
| Motif15 | 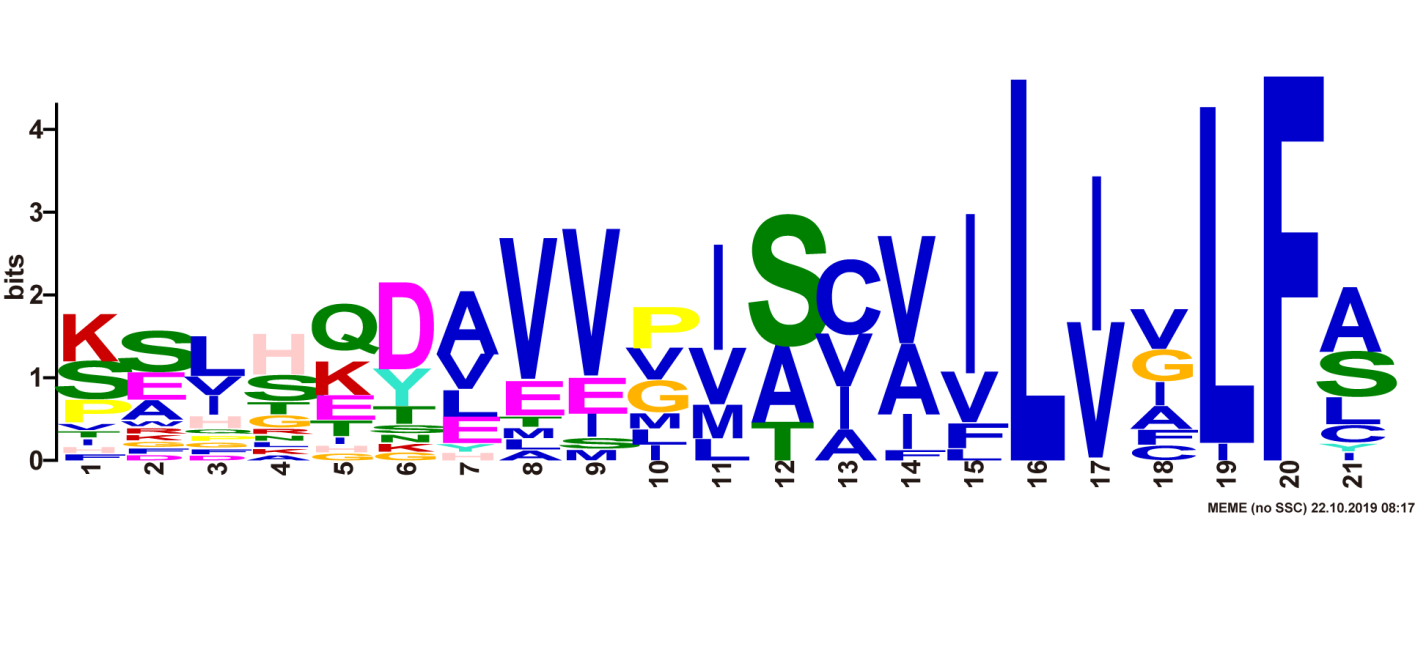 |
|  | KSLHQDAVVPISCVILIVLFA |
| Motif16 | 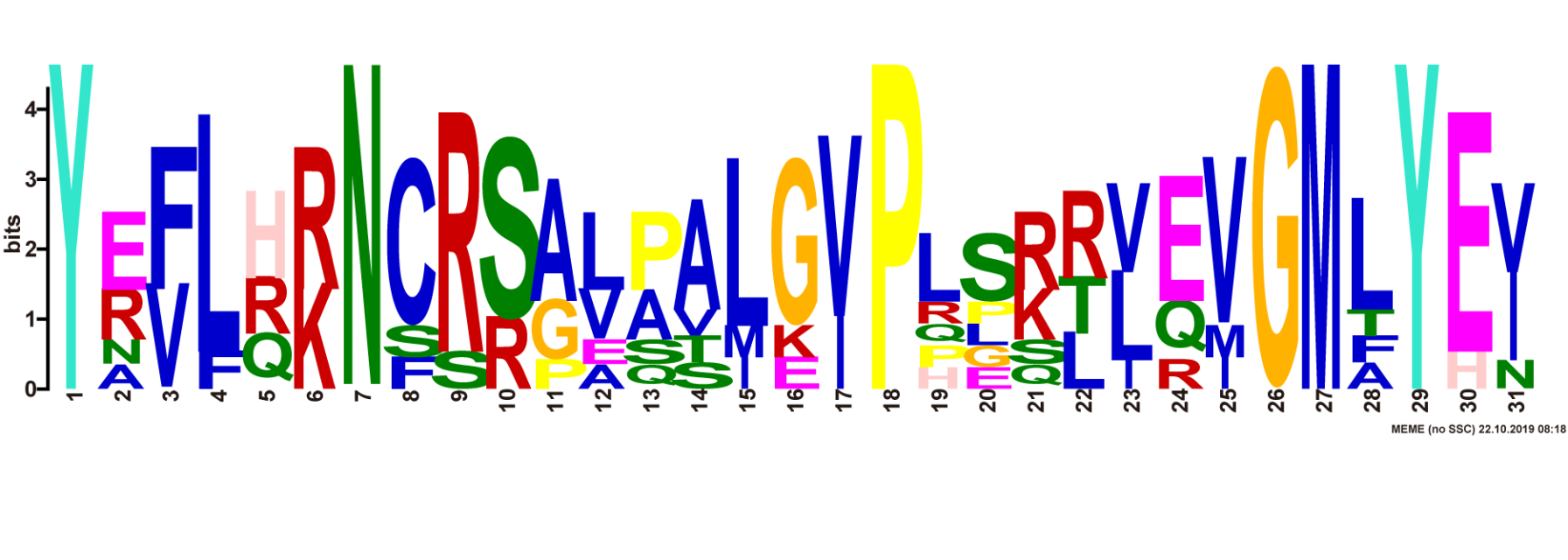   \|  \| \| --- \| |
|  | YEFLHRNCRSALPALGVPLSRRVEVGMLYEI |
| Motif17 | 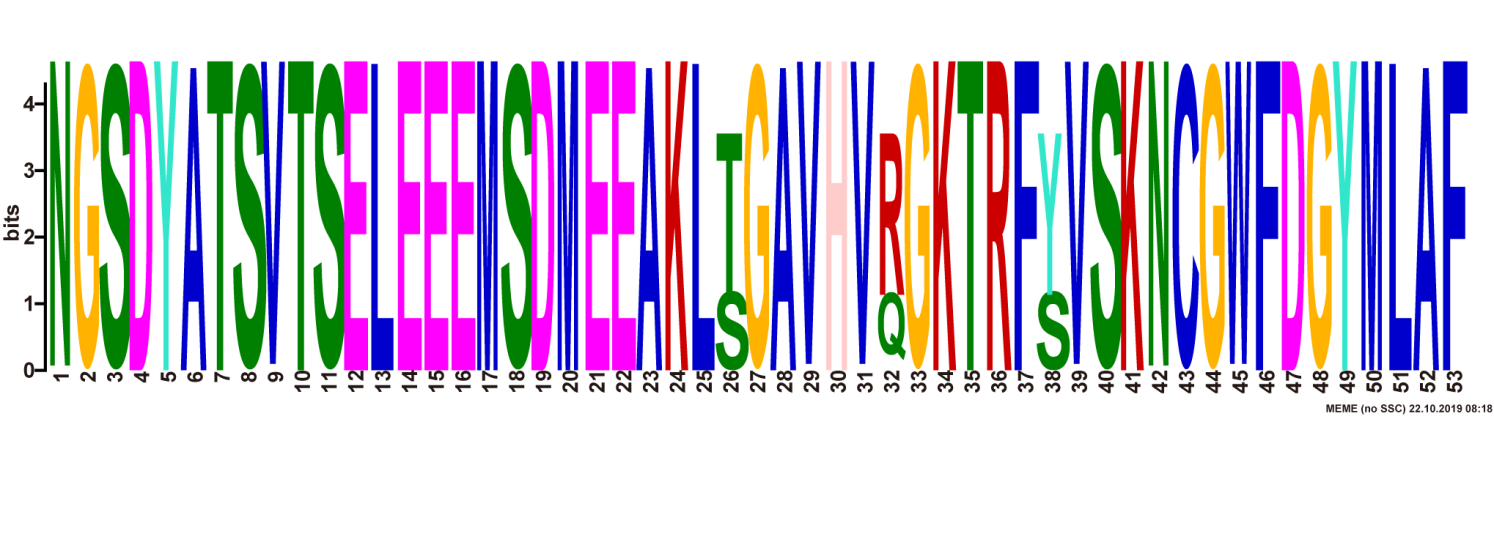 |
|  | NGSDYATSVTSELEEEMSDMEEAKLTGAVHVRGKTRFYVSKNCGWFDGYMLAF |
| Motif18 | 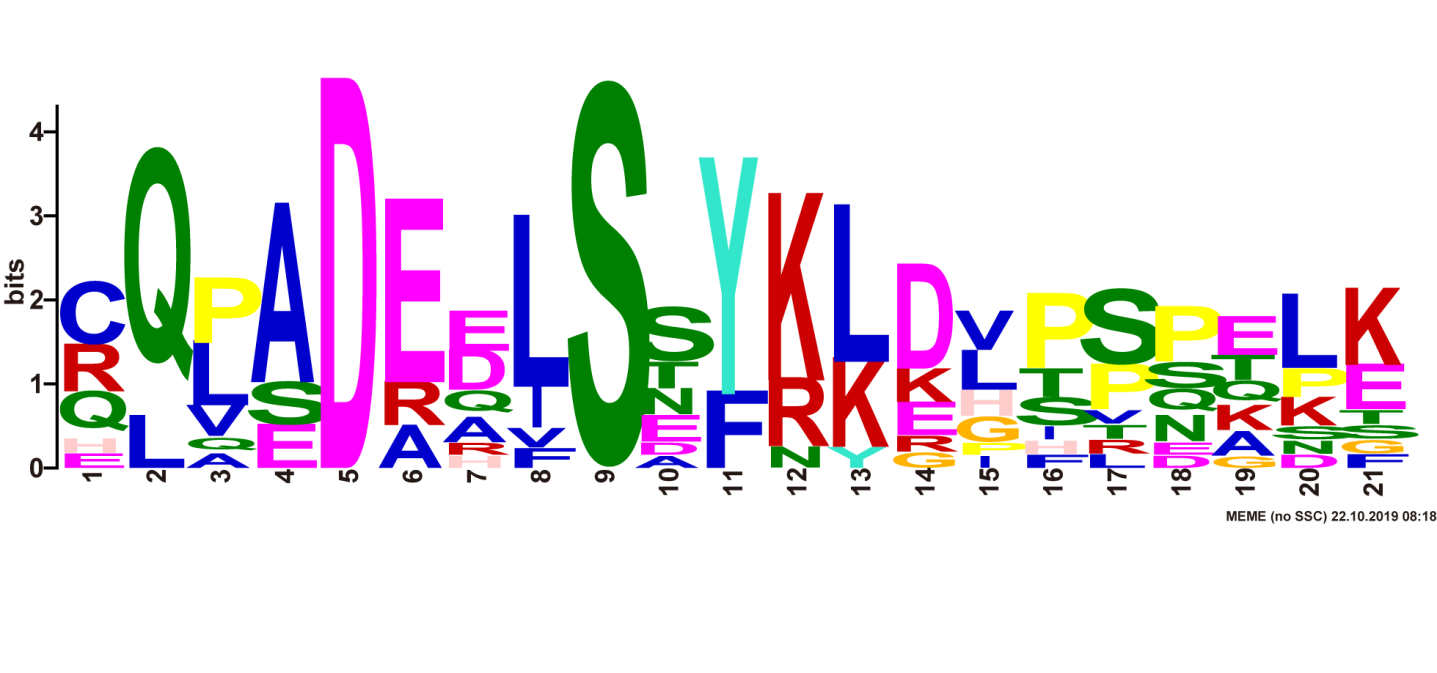 |
|  | CQPADEELSSYKLDVPSPELK |
| Motif19 | 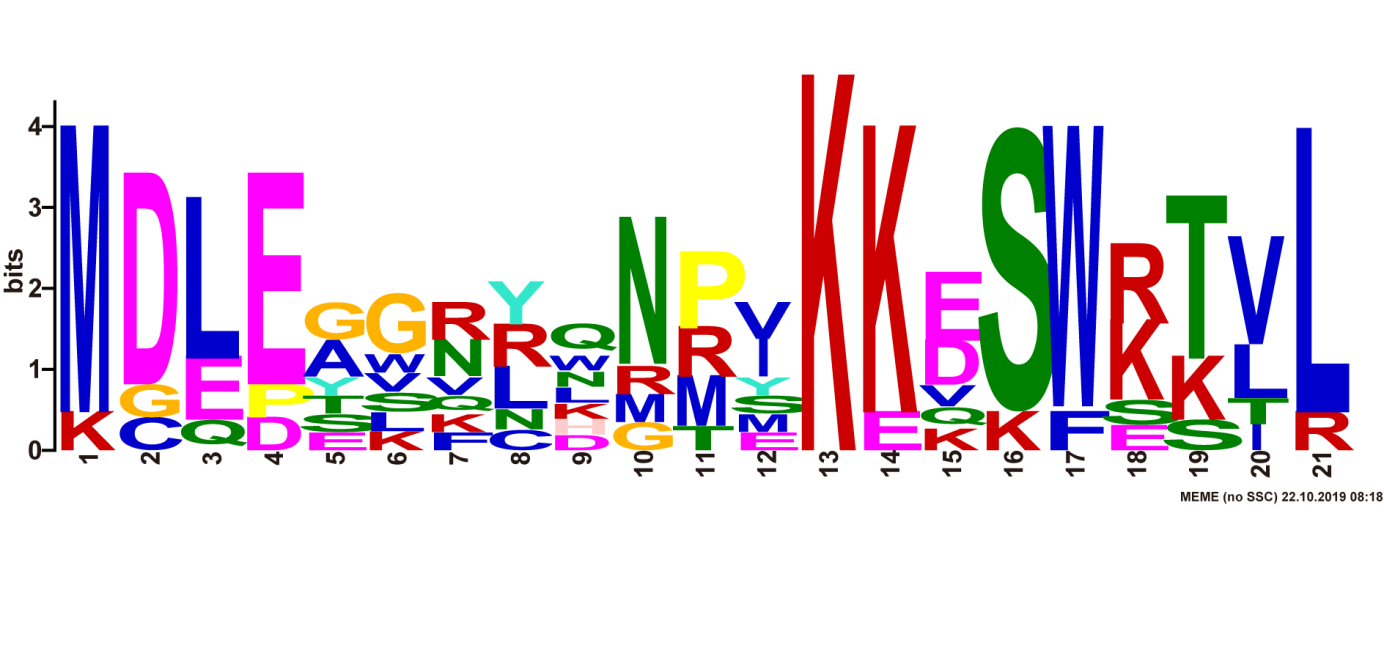 |
|  | MDLEAGRYQNPIKKESWKTVL |
| Motif20 | 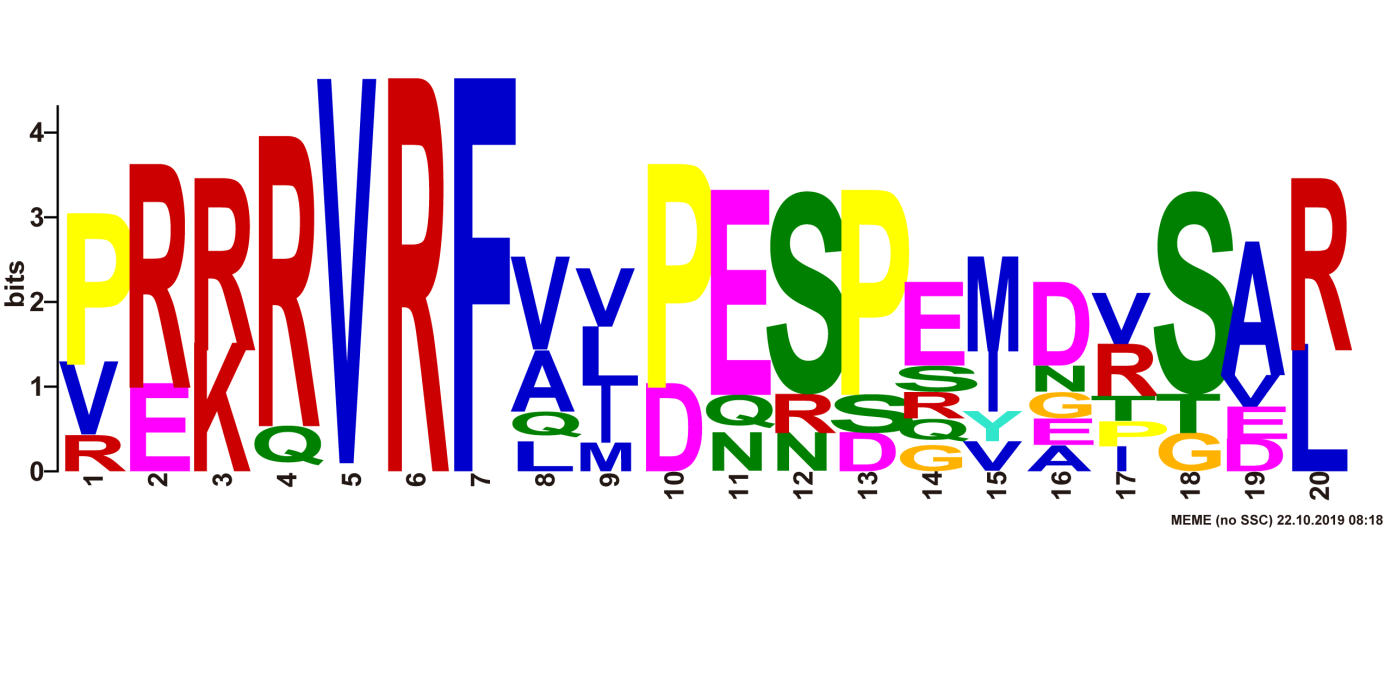 |
|  | PRRRVRFVIPESPEMDVSAR |
